# Unraveling the multifaceted roles of the *LncNAT1*-*GbCHS* module in *Ginkgo biloba* for flavonoid biosynthesis and plant development

**DOI:** 10.48130/forres-0026-0006

**Published:** 2026-03-25

**Authors:** Sian Liu, Yinlong Shuai, Peng Wan, Meng Cao, Hanyue Zhang, Changquan Zhang, Xi Zhang, Lingyu Ma, Biao Jin, Li Wang

**Affiliations:** 1 College of Horticulture and Landscape, Yangzhou University, Yangzhou 225009, China; 2 College of Agriculture, Yangzhou University, Yangzhou 225009, China; 3 State Key Laboratory of Tree Genetics and Breeding, National Engineering Research Center of Tree Breeding and Ecological Restoration, Key Laboratory of Genetics and Breeding in Forest Trees and Ornamental Plants, Ministry of Education, College of Biological Sciences and Technology, Beijing Forestry University, Beijing 100083, China; 4 Research Institute of Wood Industry, Chinese Academy of Sciences, Beijing 100091, China

**Keywords:** Flavonoids, Chalcone synthase, *Ginkgo biloba*, *LncNAT1*-*GbCHS* module, Plant development

## Abstract

Flavonoids are the primary bioactive compounds in *Ginkgo biloba* leaves. However, the regulatory mechanisms underlying their biosynthesis remain incompletely understood. In this study, three *G. biloba* exhibiting significant differences in flavonoid content were selected from a total of 26 cultivars. Integrated metabolomic, lncRNA, and mRNA sequencing analyses identified *GbCHS* as a core gene involved in flavonoid biosynthesis. Overexpression of *GbCHS* markedly enhanced flavonoid accumulation, whereas virus-induced gene silencing (VIGS) of *GbCHS* resulted in a significant reduction in total flavonoid content, confirming its essential role in flavonoid biosynthesis. Notably, *GbCHS*-silenced *G. biloba* plants also exhibited a significant decrease in plant height, leaf weight, root length, and lateral root number. In contrast, *Arabidopsis thaliana* plants overexpressing *GbCHS* showed significant increases in plant height, leaf weight, root length, and lateral root number, indicating that this gene also promotes plant development. Furthermore, *GbCHS* was found to be partially complementary to the lncRNA *LncNAT1*. Overexpression of *LncNAT1* in *G. biloba* calli significantly suppressed *GbCHS* expression and reduced total flavonoid content, whereas silencing *LncNAT1* led to increased *GbCHS* expression, flavonoid accumulation, plant height, and leaf weight. Mechanistically, *LncNAT1* represses *GbCHS* expression through the formation of an *LncNAT1*-*GbCHS* RNA duplex. Collectively, these findings reveal the multidimensional regulatory functions of the novel *LncNAT1*-*GbCHS* module in flavonoid biosynthesis and plant development in *G. biloba*.

## Introduction

Flavonoids are a class of specialized metabolites widely distributed throughout the plant kingdom, serving an essential role in both plant defense and human health owing to their distinctive chemical structures and diverse biological activities^[1,2]^. In plants, flavonoids provide protection against a broad range of biotic and abiotic stresses, including ultraviolet radiation, low temperature, drought, salinity, and pest attack^[3−9]^. In addition to their ecological functions, flavonoids exhibit notable pharmacological properties, such as antioxidant, antimicrobial, cardioprotective, and neuroprotective activities^[10]^. The flavonoid biosynthetic pathway responsible for flavonoid biosynthesis is evolutionarily conserved across plant species and relies on the coordinated action of several key enzymes, including chalcone synthase (CHS), chalcone isomerase (CHI), and flavonol synthase (FLS)^[11]^. Among these, CHS acts as the first rate-limiting enzyme and has therefore been extensively investigated in diverse plant taxa, such as maize, sorghum, petunia, orchids, and *A. thaliana*^[12]^. Functional studies in non-model plants have further validated the central role of *CHS* in flavonoid accumulation. For instance, heterologous expression of *ScCHS1* from *Stenoloma chusanum* partially complemented the anthocyanin-deficient phenotype of the *A. thaliana* *tt4* mutant^[13]^. Similarly, overexpression of *CHS* in *Glycyrrhiza uralensis* and *Camellia sinensis* resulted in significant increases in flavonoid content^[14,15]^. Conversely, suppression of *CHS* expression via RNA interference and related approaches markedly impairs flavonoid biosynthesis and leads to visible pigmentation changes. In Chinese cabbage, silencing *BrCHS4* induced a transition from purple to green leaves and was accompanied by substantial reductions in flavonoid and anthocyanin levels^[16]^. Collectively, these findings highlight the indispensable role of *CHS* in flavonoid biosynthesis and provide a theoretical basis for the genetic manipulation of plant secondary metabolism.

Despite these advances, several challenges remain. Although considerable progress has been made in deciphering the transcriptional regulation of *CHS* genes, particularly through the identification of transcription factors such as *MYB* and *bHLH* that directly activate *CHS* expression, the regulatory roles of non-coding RNAs remain largely unexplored^[17−19]^. Recent evidence indicates that 22-nt small interfering RNAs (siRNAs) in soybean regulate seed coat color by targeting *CHS* genes^[20]^. However, the involvement of long non-coding RNAs (lncRNAs) or other non-coding RNA types in this process remains unclear. Furthermore, accumulating evidence suggests that *CHS* exhibits functional versatility beyond flavonoid biosynthesis. In addition to its metabolic role, *CHS* has been reported to negatively regulate endogenous auxin transport and to influence developmental processes such as floral organ morphogenesis^[21,22]^. Collectively, these observations imply that *CHS* may participate in a diverse physiological process through complex regulatory circuits, highlighting the necessity for a more comprehensive investigation of its regulatory networks and functional breadth.

The regulatory functions of lncRNAs in flavonoid biosynthesis are gradually being elucidated, though existing studies have mainly centered on indirect regulatory mechanisms. As an illustration, studies in apple have shown that the light-induced transcription factor *MdWRKY1* binds to the promoter of *MdLNC409* and subsequently modulates *MdERF109* via this lncRNA to enhance anthocyanin biosynthesis^[23]^. Similarly, another light-induced lncRNA, *MdLNC610*, enhances the promoter activity of *MdACO1*, leading to increased ethylene production and elevated anthocyanin accumulation^[24]^. A particularly prevalent regulatory paradigm involves endogenous target mimics, in which lncRNAs function as competitive decoys for microRNAs (miRNAs), indirectly modulating the expression of downstream target genes. In sea buckthorn, for example, *LNC1* and *LNC2* act as endogenous target mimics by sequestering miR156a and miR828a, respectively^[25]^. A comparable mechanism has been described in apple, where the lncRNAs *MLNC3.2* and *MLNC4.6* bind miR156a, thereby blocking its cleavage of the target genes *SPL2* and *SPL33*. The resulting *SPL2/33* proteins subsequently form a transcriptional activation complex with the key anthocyanin regulator *MdMYB1*, which binds to the *MdCHS* promoter and activates the expression of downstream structural genes such as *MdDFR* and *MdUFGT*, ultimately enhancing light-induced anthocyanin accumulation^[26]^. By contrast, direct regulatory roles of lncRNA in flavonoid biosynthesis appear to be rare. One notable example is the intergenic *LINC15957* in radish, which has been proposed to promote anthocyanin accumulation through the regulation of transcription factors; nevertheless, its precise target genes and molecular mechanism remain unresolved^[27]^. Taken together, existing studies predominantly demonstrated that intergenic lncRNAs regulate flavonoid biosynthesis indirectly, most commonly via endogenous target mimicry or transcriptional modulation. Whether antisense lncRNAs (lncNATs) can directly regulate flavonoid biosynthesis by physically interacting with structural genes such as *CHS* has not yet been reported.

As a living fossil plant with a distinctive evolutionary history, *G. biloba* also possesses substantial economic value. Extracts originating from *G. biloba* leaves are widely used in the prevention and treatment of cerebrovascular disorders and also show therapeutic potential in the clinical management of neurodegenerative disorders^[28]^. Among the bioactive constituents of *G. biloba* extracts, flavonoids represent the most abundant and pharmacologically valuable components. Consequently, characterizing the core genes that govern flavonoid biosynthesis in *G. biloba* is critically important for enhancing flavonoid accumulation and improving resource utilization. To date, numerous structural enzyme genes involved in flavonoid biosynthesis, as well as key regulatory factors such as *MYB*, *bHLH*, and *bZIP* transcription factors, have been identified in *G. biloba* through high-throughput sequencing^[29−32]^. However, the functional validation of these genes and the demonstration of their precise regulatory mechanisms in flavonoid biosynthesis remain inadequately explored. Furthermore, transcriptomic analyses of *G. biloba* leaves have suggested that lncRNAs may influence flavonoid biosynthesis by modulating the expression of transcription factors or flavonoid biosynthesis genes^[33]^. More recently, an integrative analysis combining PacBio third-generation and Illumina second-generation sequencing across eight *G. biloba* tissues predicted that five lncRNAs are potentially involved in the regulation of flavonoid biosynthesis^[34]^. However, direct experimental evidence demonstrating whether these lncRNAs can physically interact with flavonoid biosynthetic enzyme genes and directly modulate their function remains lacking.

In this present study, in order to identify key enzyme genes and lncRNAs associated with flavonoid biosynthesis in *G. biloba*, the study surveyed 26 cultivars and screened for individuals exhibiting high and low flavonoid contents. Leaves from three cultivars displaying pronounced differences in flavonoid accumulation were subsequently selected for integrated metabolomic and strand-specific RNA sequencing (ssRNA-seq) analyses. Through comprehensive data integration, a flavonoid-related *LncNAT1*-*GbCHS* regulatory module was identified. *LncNAT1* is a type of lncNAT that partially overlaps with the *GbCHS* gene and suppresses its expression through sequence-specific binding, thereby negatively regulating flavonoid biosynthesis. In addition, the results demonstrate that GbCHS functions not only as a key enzyme in flavonoid biosynthesis but also plays broader roles in plant growth and development, including the regulation of lateral root formation, branching architecture, and leaf production. Collectively, this study reveals a previously unrecognized mechanism by which an lncNAT regulates flavonoid biosynthesis through its interaction with a structural enzyme gene and uncovers a novel developmental function of GbCHS. These findings provide valuable genetic insights for the improvement of *G. biloba* leaf yield and flavonoid accumulation in future breeding programs.

## Materials and methods

### Plant materials and growth conditions

Plant materials were obtained from a *G. biloba* germplasm resource nursery located in Pizhou, Jiangsu Province, China. Leaves from 26 *G. biloba* cultivars of comparable age and grown under uniform environmental conditions were collected from the nursery. Detailed information for each cultivar is provided in Supplementary Table S1. Among these, leaves from the cultivars designated 'DHS', 'DTH' and 'HBAL' were selected for subsequent metabolomic profiling and high-throughput sequencing analyses.

### Determination of flavonoids and flavonols

Flavonol compounds were quantified using high-performance liquid chromatography (HPLC) following a previously described protocol^[33]^. In addition, total flavonoid content was determined using a commercial plant flavonoid assay kit.

### Widely targeted metabolic profiling

Freeze-dried leaf samples were ground into a fine powder, and 100 mg of each sample was extracted with 70% (v/v) aqueous methanol. The extracts were incubated at 4 °C overnight, with intermittent vortexing to enhance extraction efficiency. Widely targeted metabolomic profiling was performed using ultra-performance liquid chromatography coupled with tandem mass spectrometry (UPLC-MS/MS), as described previously^[35]^. Differentially accumulated metabolites (DAMs) were identified based on a variable importance in projection (VIP) ≥ 1 and a fold change ≥  2 or ≤  0.5.

### lncRNA identification and ssRNA-seq

Total RNA was extracted from leaf tissues of the three selected cultivars ('DHS', 'DTH', and 'HBAL') using the RNAprep Pure Plant Kit following the manufacturer’s protocol. A total of nine strand-specific cDNA libraries were constructed and subjected to high-throughput sequencing. Clean reads obtained from each library were aligned to the *G. biloba* genome using Hisat2, followed by transcript assembly with StringTie^[36]^. LncRNAs were identified using an established pipeline^[33]^, which included filtering based on transcript length, annotation status, and coding potential. This potential was assessed using CPC2, Pfam, and CNCI^[37,38]^.

### Differential expression analysis of lncRNAs and mRNAs

Differentially expressed lncRNAs (DELs) and mRNAs (DEGs) were identified using the DESeq2 package^[39]^, with an adjusted *p*-value < 0.05 and an absolute log_2_ fold change > 1 set as the significance thresholds. Functional enrichment analysis was subsequently conducted, with GO enrichment performed using the GOseq tool^[40]^, while KEGG pathway enrichment was assessed using the clusterProfiler 4.0 software^[41,42]^.

### Gene cloning, vector construction, and GbCHS enzyme activity assays

The full-length sequences of *LncNAT1* and the coding sequence (CDS) of *GbCHS* were amplified and cloned into the overexpression vector pRI101 through the ClonExpress II One Step Cloning Kit. Using the same strategy, *GbCHS* was also inserted into the *pACT2-LUC* and *pET-32a* vectors. For enzymatic characterization, the *pET-32a-GbCHS* construct was transformed into *Escherichia coli* BL-21 (DE3) cells, and recombinant protein expression was induced with 0.2 mM IPTG at 16 °C for 16 h. The resulting fusion protein was purified following a reported protocol^[43]^ and verified by SDS-PAGE. Enzyme activity assays were conducted by incubating purified GbCHS in a reaction mixture comprising 160 μM p-coumaroyl-CoA, 480 μM malonyl-CoA, and 0.1 M potassium phosphate buffer. Three different amounts of purified protein (120, 150, and 180 μg) were assessed individually at 30 °C for 1 h. After incubation, reaction mixtures were extracted twice with ethyl acetate, centrifuged, evaporated under nitrogen gas, and resuspended in methanol. Heat-inactivated pET-32a-GbCHS protein was used as a negative control.

### qRT-PCR analysis

For qRT-PCR analysis, *GAPDH* served as an internal control gene^[30]^. Gene-specific primers were designed with Primer Premier 5.0 and are listed in the Supplementary Table S2. Amplification was performed on a CFX96™ Real-Time PCR System using ChamQ SYBR qPCR Master Mix. Relative expression levels were calculated using the 2^−ΔΔCᴛ^ method^[44]^.

### Genetic transformation of *A. thaliana* plants and *G. biloba* calli

The *35S::GbCHS* construct was first introduced into *Agrobacterium tumefaciens* strain GV3101 and used to transform *A. thaliana* via the floral dip method. Seeds harvested from transformed plants were germinated on kanamycin-containing medium, and PCR analysis was performed to confirm transgene integration. PCR-positive transgenic plants were selected for subsequent analyses, including gene expression assessment, phenotypic observation, and determination of flavonoid content. Additionally, the *35S::LncNAT1* and *35S::GbCHS* vectors were transformed into *G. biloba* calli following a previously described method^[45]^. Transgenic calli were used for gene expression analysis and flavonoid quantification.

### Genetic transformation of *G. biloba* calli

The *35S::LncNAT1* and *35S::GbCHS* constructs were introduced into *A. tumefaciens* strain EHA105 and subsequently used to infect *G. biloba* calli according to established protocols^[29]^.

### Virus-induced gene silencing (VIGS) transformation

Specific 300-bp fragments of the *LncNAT1* and *GbCHS* sequences were amplified using designed primers (Supplementary Table S2) and cloned into the TRV vector. The resulting constructs were transformed into *A. tumefaciens* strain GV3101 and used for VIGS in seedlings following previously reported procedures^[45]^.

### Dual-luciferase reporter assay and RNase protection assay

For the dual-luciferase reporter (DLR) assays, protoplasts were isolated from poplar leaves as previously described^[46]^. Different plasmid combinations of effector and reporter plasmids, *pRI101-GFP* with GbCHS-LUC, or *35S::LncNAT1* with GbCHS-LUC, were co-transferred into these protoplasts. Firefly luciferase (LUC) and Renilla luciferase (REN) activities were quantified with a GLO-MAX® 20/20 Luminometer. In parallel, the same effector and reporter constructs were co-infiltrated into leaves of *Nicotiana benthamiana*, and luminescence signals were visualized using a Tanon-5200 *in vivo* plant imaging system.

RNase protection assay (RPA) was performed as previously described^[47]^. Briefly, total RNA extracted from *G. biloba* tissues was treated with RNase A in a digestion buffer to degrade single-stranded RNA. The remaining RNA was subjected to RT-PCR and agarose gel electrophoresis to evaluate the formation of the *LncNAT1*-*GbCHS* duplex and its protective effect against RNase degradation.

### Measurement of photosynthetic rate

Photosynthetic parameters of rosette leaves from *A. thaliana* plants were measured using a portable gas-exchange system. Measurement conditions were set to 24 °C, a photosynthetically active radiation of 1,100 μmol photons/(m^2^·s), an ambient CO_2_ of 400 μmol/mol, and a relative humidity of 65%. Five biological replicates (one plant per replicate) were analyzed for each genotype.

## Results

### Flavonoid content exhibits significant variation among *G. biloba* cultivars

**Figure 1:**
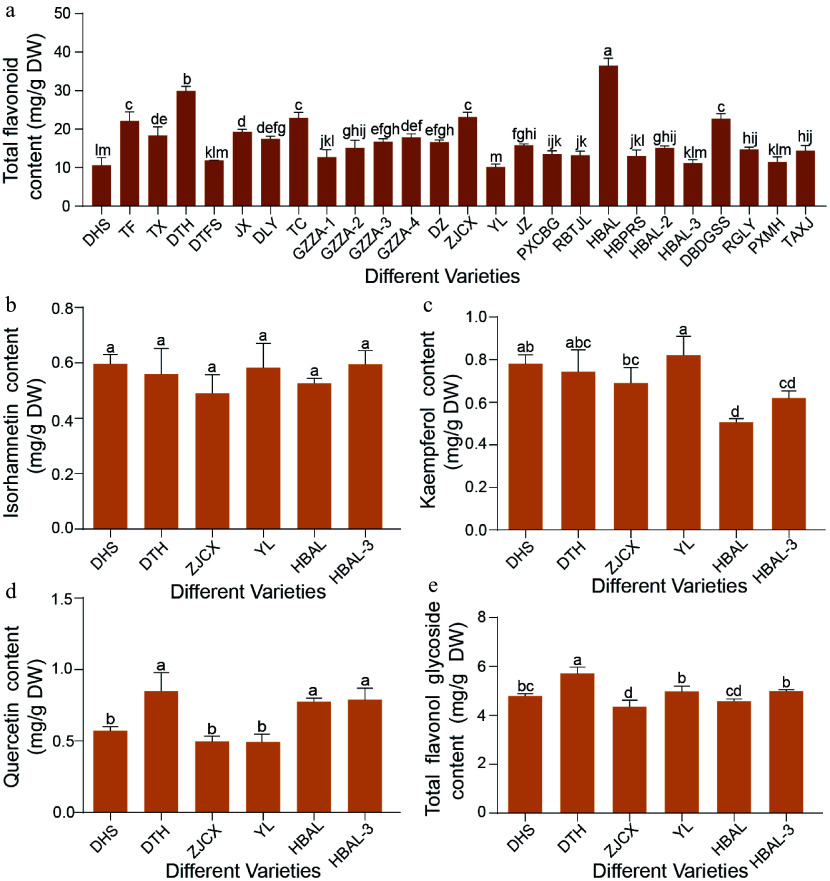
Variation in flavonoid content among different *G. biloba* cultivars. (a) Total flavonoid levels in leaves of 26 *G. biloba* cultivars. (b) Isorhamnetin, (c) kaempferol, (d) quercetin, and (e) total flavonol glycoside contents in leaves of six selected cultivars. Statistical significance was determined by one-way ANOVA. Different lowercase letters indicate significant differences (*p < 0.05*). Data are presented as mean ± SD (*n* = 3 biological replicates).

Quantitative analysis of leaf samples from 26 *G. biloba* cultivars revealed substantial variation in total flavonoid contents (Fig. 1a). Among these cultivars, 'HBAL' exhibited the highest accumulation (36.46 mg/g), followed by 'DTH' (29.88 mg/g) and 'ZJCX' (23.14 mg/g). In contrast, 'YL' showed the lowest accumulation (10.15 mg/g), with 'DHS' (10.59 mg/g) and 'HBAL-3' (11.19 mg/g) also displaying relatively low levels. Overall, the total flavonoid content in 'HBAL' was approximately 3.45-fold higher than that in 'YL' (Fig. 1a).

Given that flavonol glycosides, including kaempferol, quercetin, and isorhamnetin, are the major bioactive constituents of *G. biloba* leaves, their contents were further quantified in six representative cultivars. Isorhamnetin content remained relatively stable across the six cultivars ('HBAL', 'DTH', 'ZJCX', 'YL', 'DHS', and 'HBAL-3') (Fig. 1b). In contrast, kaempferol content varied markedly, with 'YL' exhibiting the highest level (Fig. 1c). Quercetin content also differed significantly among cultivars, reaching a maximum of 0.85 mg/g in 'DTH' (Fig. 1d). Consistently, 'DTH' displayed the highest total flavonol glycoside content (5.71 mg/g). In contrast, the lowest quercetin content (0.57 mg/g) was observed in 'DHS', which also showed a comparatively low total flavonol glycoside level of 4.89 mg/g (Fig. 1e).

### Metabolomic analysis reveals distinct flavonoid profiles and pathway enrichment in *G. biloba* cultivars

To explore differences in flavonoid composition, leaves from three cultivars with distinct flavonoid profiles were selected: 'HBAL', which exhibited the highest total flavonoid content, 'DTH', which showed the highest flavonol glycoside accumulation, and 'DHS', which displays the lowest levels of both total flavonoids and flavonol glycosides. Totally, the analysis detected 976 metabolites, predominantly flavonoids (22.8%), phenolic acids (18.2%), lipids (17.1%), amino acids and their derivatives (9.2%), organic acids (7.4%), and other compound classes (8.4%) (Supplementary Table S3). DAM analysis identified 226 DAMs between 'DTH' and 'DHS', including 168 up-accumulated and 58 down-accumulated metabolites; 315 DAMs between 'HBAL' and 'DHS' (206 up- and 109 down-accumulated); and 181 DAMs between 'HBAL' and 'DTH' (97 up, 84 down) (Supplementary Fig. S1).

**Figure 2:**
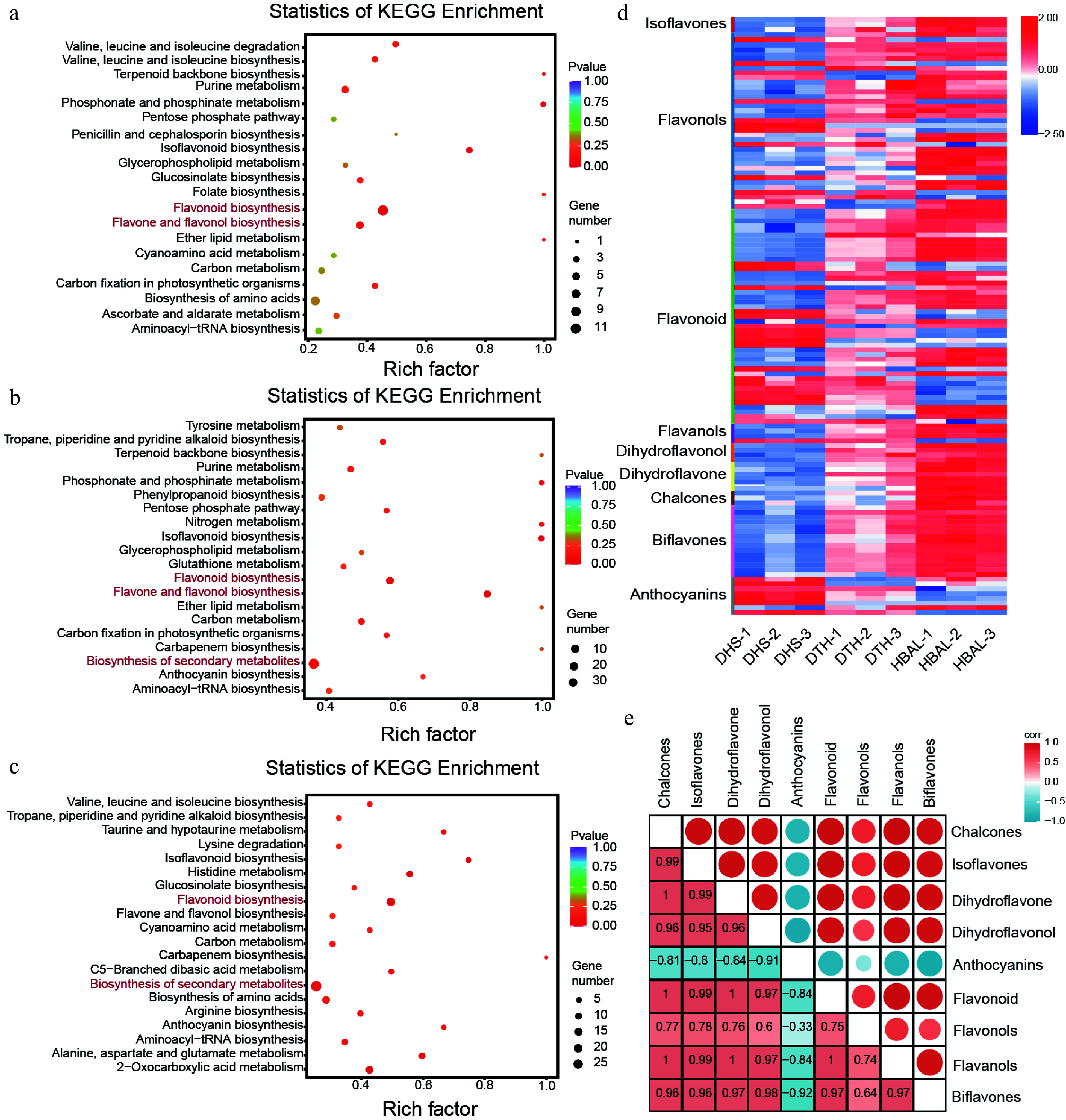
Metabolomic analysis of three *G. biloba* cultivars. KEGG pathway enrichment analysis of DAMs in the comparisons between (a) 'DTH' and 'DHS', (b) 'HBAL' and 'DHS', and (c) 'DTH' and 'HBAL'. The rich factor represents the proportion of DAMs mapped to a given pathway relative to the total number of annotated metabolites in that pathway, and the circle size indicates the number of DAMs. (d) Distribution of differentially accumulated flavonoid metabolites among cultivars. (e) Correlation analysis between anthocyanins and other flavonoid subclasses.

KEGG pathway enrichment analysis of the DAMs revealed that metabolites showing differential accumulation in all three pairwise comparisons ('DTH' vs 'DHS', 'HBAL' vs 'DHS', and 'DTH' vs 'HBAL') were predominantly enriched in pathways including flavonoid biosynthesis as well as flavone and flavonol biosynthesis pathways (Fig. 2a−c). Accordingly, further focus was placed on flavonoid-related DAMs, and a total of 125 differentially accumulated flavonoid metabolites were identified and classified into various categories: isoflavones, flavonols, flavones, dihydroflavonols, dihydroflavones, chalcones, biflavones, and anthocyanins. With the exception of anthocyanins, the majority of flavonoid subclasses exhibited higher accumulation in 'HBAL' or 'DTH' compared with 'DHS' (100%, 3/3), flavonols (73%, 27/37), flavones (69%, 31/45), flavanols (75%, 3/4), dihydroflavonols (100%, 4/4), dihydroflavones (100%, 6/6), chalcones (100%, 3/3), and biflavones (100%, 15/15) were higher in the 'HBAL' or 'DTH' cultivars compared to the 'DHS' cultivar. In contrast, 88% (7/8) of anthocyanin metabolites accumulated at higher levels in 'DHS' than in both 'DTH' and 'HBAL' (Fig. 2d, Supplementary Fig. S2). Correlation analysis further supported this divergence in flavonoid composition. Flavonols, isoflavones, flavones, dihydroflavonols, dihydroflavones, chalcones, and biflavones exhibited strong positive correlations with one another, whereas anthocyanins showed a negative correlation with these flavonoid subclasses (Fig. 2e).

### Integration of transcriptome and metabolome identifies key genes in flavonoid biosynthesis of *G. biloba*

ssRNA-seq was conducted on leaf tissues from the three cultivars exhibiting marked differences in flavonoid accumulation. In total, 3,090 DEGs were identified across all pairwise comparisons. Specifically, 689 DEGs (318 upregulated, 371 downregulated) were identified in the 'DTH' vs 'DHS' comparison, while 'HBAL' vs 'DHS' showed 2,195 DEGs (1,418 upregulated, 777 downregulated), and 'DTH' vs 'HBAL' contained 1,545 DEGs (485 upregulated, 1,060 downregulated) (Supplementary Fig. S3). Additionally, there were 249 shared DEGs between the 'DTH' vs 'DHS' and 'DTH' vs 'HBAL' comparison groups, 300 shared DEGs between the 'DTH' vs 'DHS' and 'HBAL' vs 'DHS' comparison groups, 834 shared DEGs between the 'DTH' vs 'HBAL' and 'HBAL' vs 'DHS' comparison groups, and 44 DEGs consistently present across all three comparison sets (Supplementary Fig. S4). Integrative analysis of DEGs and DAMs revealed significant enrichment in pathways related to flavonoid biosynthesis as well as flavone and flavonol biosynthesis pathways (Supplementary Figs S5−S7).

**Figure 3:**
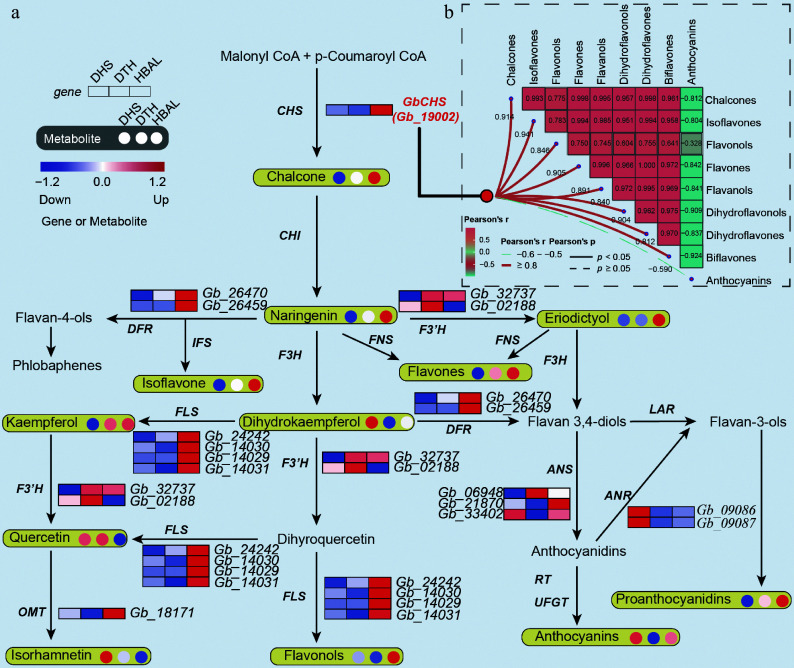
Integrated transcriptomic and metabolomic profiling of flavonoid biosynthesis in *G. biloba*. (a) Expression patterns of DEGs and accumulation levels of DAMs associated with flavonoid biosynthesis. (b) Correlation network illustrating relationships between *GbCHS* expression and different flavonoid subclasses.

Further examination identified 15 DEGs encoding key enzymes in the flavonoid biosynthesis pathway, including one *GbCHS* gene (*Gb_19002*), two *GbDFR* genes (*Gb_26470*, *Gb_26459*), two *GbANR* genes (*Gb_09086*, *Gb_09087*), two *GbF3'H* genes (*Gb_32737*, *Gb_02188*), four *GbFLS* genes (*Gb_24242*, *Gb_14030*, *Gb_14029*, *Gb_14031*), one *GbOMT* gene (*Gb_18171*), and three *GbANS* genes (*Gb_06948*, *Gb_21870*, *Gb_33402*). Except for one *GbANS* gene (*Gb_33402*) and two *GbANR* genes, the remaining genes exhibited higher expression levels in the high-flavonoid cultivar 'HBAL' or the high-flavonol cultivar 'DTH' (Fig. 3a). Transcript-metabolite correlation analysis further demonstrated that the expression profiles of *GbCHS* (*Gb_19002*), *GbF3'H* (*Gb_32737*, *Gb_02188*), and *GbFLS* (*Gb_24242*, *Gb_14030*, *Gb_14029*, *Gb_14031*) were closely aligned with the accumulation patterns of their corresponding flavonoid products (Fig. 3a).

Given that CHS is the first rate-limiting enzyme in the flavonoid biosynthesis pathway, further focus was placed on the *CHS* gene family in *G. biloba.* A total of eight *GbCHS* genes were identified in the *G. bilob*a genome, and multiple sequence alignment revealed a high degree of conservation among these genes (Supplementary Fig. S8). Expression analysis showed that only three *GbCHS* genes (*Gb_20355*, *Gb_19002*, and *Gb_01519*) were expressed at relatively high levels, whereas the remaining genes exhibited low transcript abundance (Supplementary Fig. S9). Notably, among these three, only *Gb_19002* (*GbCHS*) was significantly differentially expressed. Its transcript level in the high-flavonoid cultivar 'HBAL' was approximately 15.5-fold higher than that in the low-flavonoid cultivar 'DHS', prompting its selection for subsequent functional analyses. Correlation analysis between *GbCHS* expression and flavonoid metabolites further revealed strong positive associations with chalcones, isoflavones, dihydroflavones, dihydroflavonols, flavones, flavonols, and biflavonoids, whereas a negative correlation was observed with anthocyanins (Fig. 3b). Collectively, these results indicate that *GbCHS* (*Gb_19002*) is a central regulatory gene influencing the biosynthesis of multiple flavonoid subclasses in *G. biloba*.

### *GbCHS* serves as a pivotal structural gene for flavonoid biosynthesis

**Figure 4:**
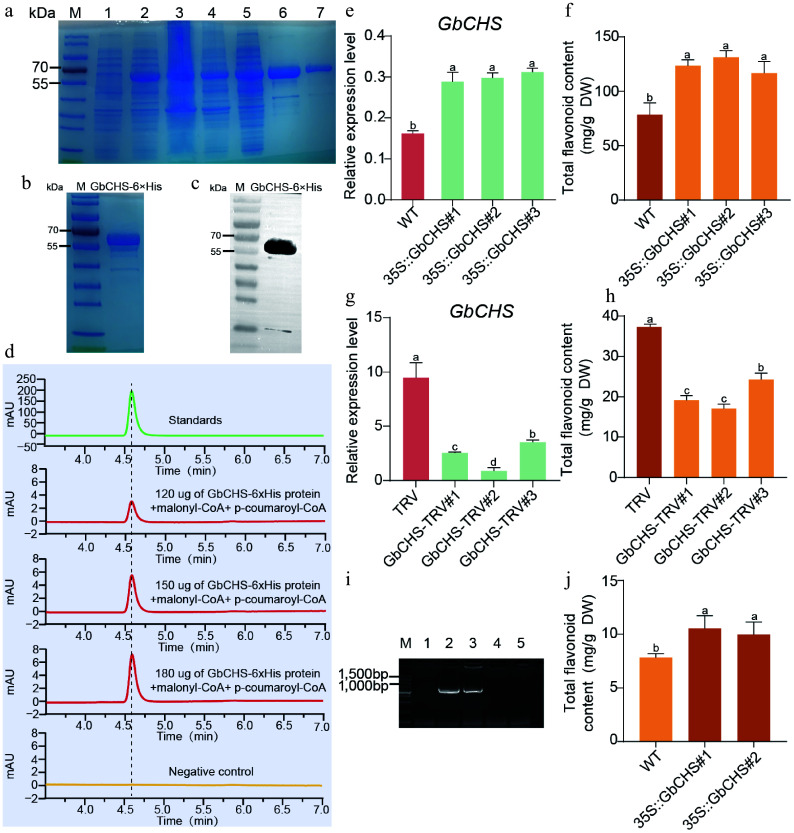
*GbCHS* promotes the accumulation of total flavonoids. (a) Expression and purification of the recombinant GbCHS protein. M: Protein marker; 1: non-induced sample; 2: induced sample; 3: supernatant after induction and lysis; 4: pellet after induction and lysis; 5: flow-through during purification; 6−7: eluted GbCHS recombinant protein. (b) Buffer exchange and quality assessment of purified GbCHS protein. (c) Western blot analysis of recombinant GbCHS protein. (d) Enzymatic activity assay of GbCHS based on naringenin production. (e) Relative expression levels of *GbCHS* in overexpressing *G. biloba* calli. (f) Total flavonoid content in *GbCHS*-overexpressing *G. biloba* calli. (g) *GbCHS* expression levels in VIGS-mediated silenced *G. biloba* plants. (h) Total flavonoid content in *GbCHS*-silenced *G. biloba* plants. (i) Positive detection of *GbCHS*-overexpressing *A. thaliana* lines. M: DNA molecular weight marker; 1: wild-type *A. thaliana* (WT); 2−3: *GbCHS*-overexpressing *A. thaliana*; 4−5: negative control. (j) Total flavonoid content in *GbCHS*-overexpressing *A. thaliana* plants. Different lowercase letters indicate significant differences (*p < 0.05*). Data are presented as mean ± SD of three biological replicates (*n* = 3).

The *GbCHS* gene, containing a CDS of 1,191 bp, was successfully cloned. In order to investigate the catalytic properties of the GbCHS enzyme, a 6×His tag was added to its N-terminus, and the recombinant protein was heterologously expressed in *E. coli* (Fig. 4a). SDS–PAGE and Western blot analyses confirmed the successful expression and purification of GbCHS, yielding a protein with an apparent molecular weight of approximately 60 kDa (Fig. 4b, c). The catalytic activity of GbCHS was evaluated using purified recombinant protein in the presence of p-coumaroyl-CoA and malonyl-CoA. The resulting reaction products were analyzed by HPLC. As chalcone products are known to undergo spontaneous cyclization to naringenin during the *in vitro* reaction process, naringenin was detected in the reaction product. Compared with the heat-inactivated control, a distinct peak corresponding to naringenin appeared at a retention time of 4.57 min in reactions containing active GbCHS. Moreover, naringenin accumulation increased proportionally with the increased amount of GbCHS protein (Fig. 4d), confirming that GbCHS is a catalytically active chalcone synthase.

In order to further assess the biological function of *GbCHS*, *A. tumefaciens,* carrying the *35S::GbCHS* vector, was transformed into *G. biloba* calli. Transcript levels were quantified using qRT-PCR. Overexpression of *GbCHS* led to a marked increase in total flavonoid content in the *G. bilob*a calli, which rose by 49% to 68% relative to the control group (Fig. 4e, f). Conversely, VIGS of *GbCHS* in *G. biloba* seedlings led to a pronounced reduction in flavonoid accumulation, with silenced lines showing 35%-55% lower total flavonoid content compared with plants transformed with the empty TRV vector (Fig. 4g, h). To further validate these findings in a heterologous system, this study generated two independent *A. thaliana* transgenic lines expressing *35S::GbCHS* (Fig. 4i, Supplementary Fig. S10). Consistent with the results observed in *G. biloba*, total flavonoid content in these transgenic *A. thaliana* lines increased significantly by 36% and 28%, respectively, relative to wild-type plants (Fig. 4j).

### *GbCHS* significantly promotes plant growth and development

**Figure 5:**
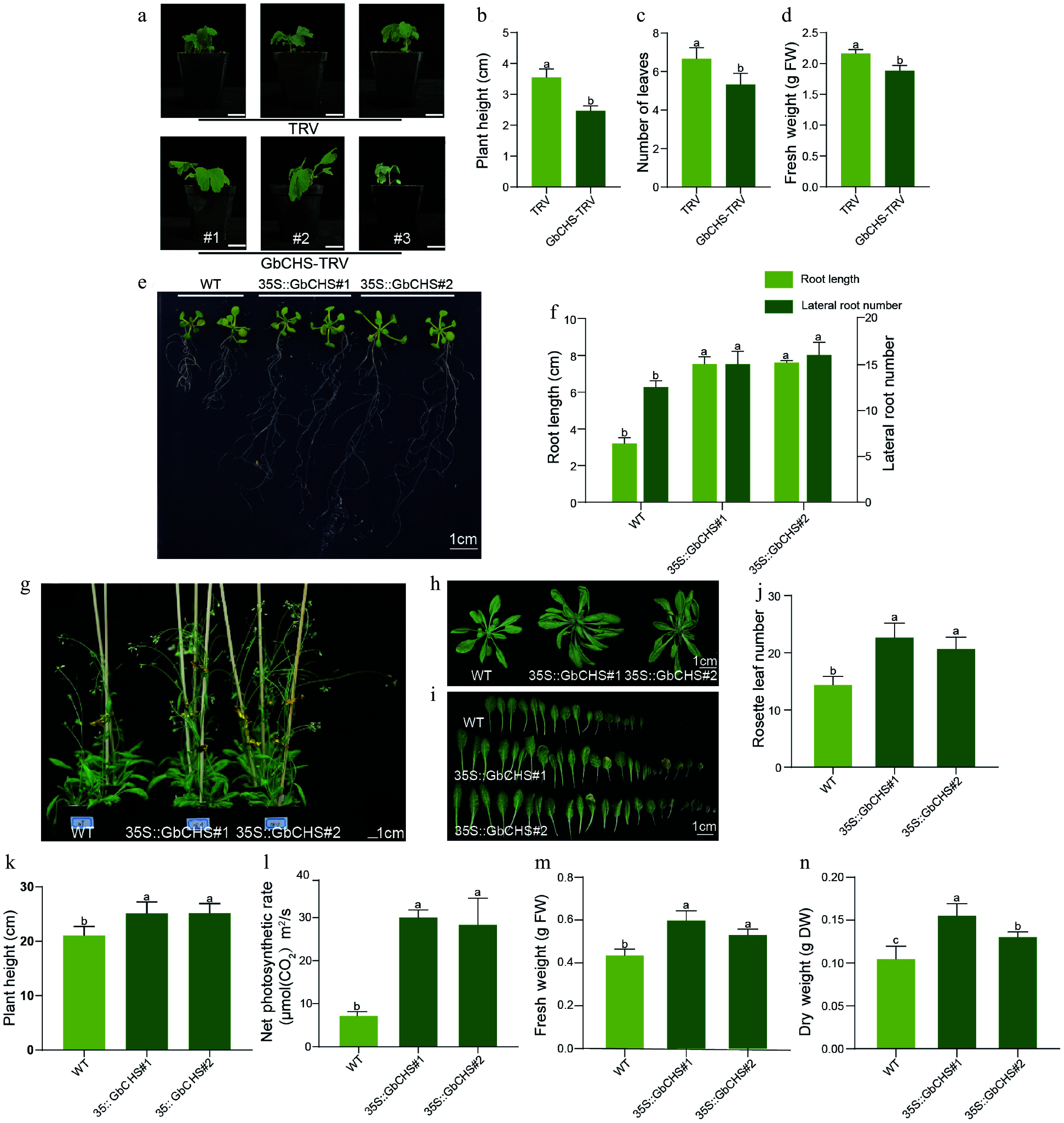
Phenotypic effects of *GbCHS* silenced in *G. biloba* and *GbCHS* overexpression in *A. thaliana* plants. (a), (b) Comparison of plant height. (c) Comparison of the number of leaves. (d) Comparison of fresh weight. Plant height was measured from the soil surface to the apical meristem. Scale bar = 3 cm. (e), (f) Comparison of root traits. (g)−(j) Comparison of rosette leaf number. (k) Comparison of plant height. (l) Comparison of net photosynthetic rate. (m) Comparison of aboveground fresh weight (FW). (n) Comparison of aboveground dry weight (DW). Statistical significance was determined by ANOVA. Different lowercase letters indicate significant differences at *p* < 0.05. All data are shown as the mean ± SD (sample sizes: *n* = 3 for panels 5B−5F; *n* = 5 for panels 5H and 5K−5P).

Silencing of *GbCHS* via VIGS markedly impaired the growth of *G. biloba*. Compared with plants transformed with the TRV empty vector, *GbCHS*-silenced *G. biloba* exhibited a 30% reduction in plant height, a 20% decrease in leaf number, and a 13% reduction in fresh weight (Fig. 5a−d). Consistently, heterologous overexpression of *GbCHS* in *A. thaliana* significantly promoted root system development. Both transgenic lines overexpressing *GbCHS* produced substantially more lateral roots than wild-type plants (Fig. 5e). Quantitative analysis revealed that *GbCHS#1* and *GbCHS#2* formed an average of 14.78 and 16.00 lateral roots, respectively, representing a 20%-30% increase compared with WT of 12.33 (Fig. 5f). In addition, primary root length was dramatically enhanced in *GbCHS*-overexpressing plants, reaching 7.51 cm in *GbCHS#1* and 7.60 cm in *GbCHS#2*, more than twice the length observed in WT plants (3.18 cm) (Fig. 5f). Aboveground growth was also strongly stimulated by *GbCHS* overexpression. The number of rosette leaves increased by 58% and 44% in the two *GbCHS*-overexpression *A. thaliana* lines, respectively, relative to WT (Fig. 5g−j), while plant height increased by 19.0% and 19.4% (Fig. 5k). Moreover, net photosynthetic rate was elevated 3.2-fold and 2.9-fold (Fig. 5l). Correspondingly, aboveground biomass accumulation was significantly enhanced, with fresh weight increasing by 38% and 22% and dry weight by 49% and 24% in the two overexpression lines (Fig. 5m, n).

### Identification of lncRNAs predicts the potential *LncNAT-GbCHS* regulatory module in *G. biloba*

**Figure 6:**
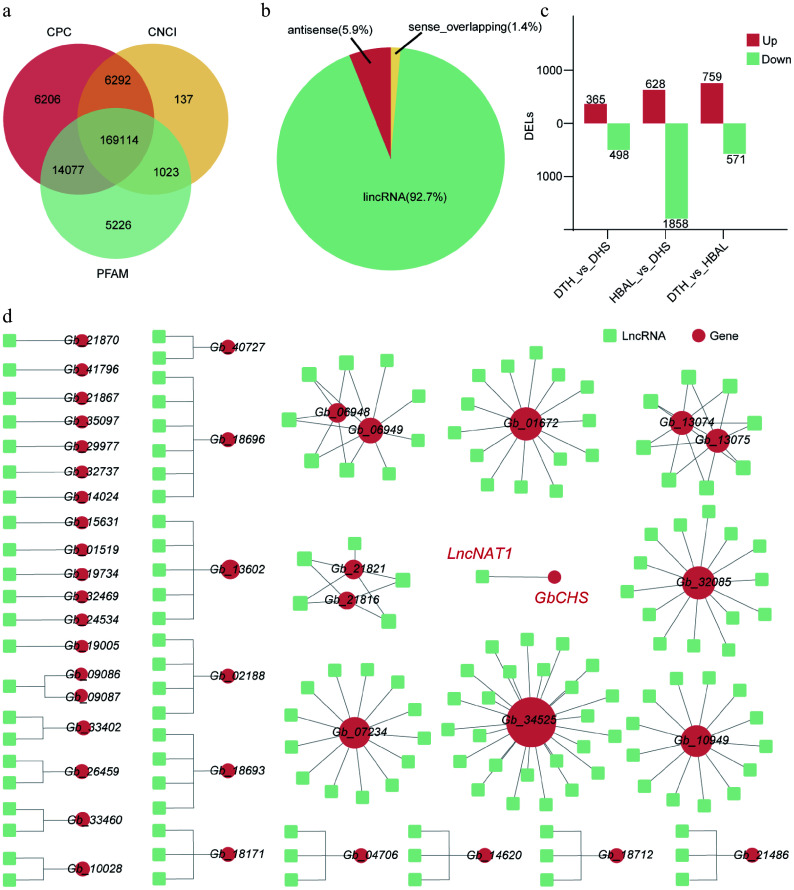
Genome-wide identification and regulatory network analysis of lncRNAs in three *G. biloba* cultivars. (a) Identification pipeline and number of lncRNAs detected. (b) Classification of identified lncRNAs into lincRNAs. (c) Numbers of DELs among the three cultivar comparisons. (d) DELs-flavonoid biosynthesis gene networks.

In order to systematically identify lncRNAs in *G. biloba*, this study assessed the protein-coding potential of newly assembled transcripts using CPC, PFAM, and CNCI. This analysis identified a total of 169,114 lncRNAs (Fig. 6a), comprising 156,761 lincRNAs (92.7%), 9,973 lncNATs (5.9%), and 2,380 sense-overlapping lncRNAs (1.4%) (Fig. 6b). Differential expression analysis revealed 3,448 DELs among the three cultivar comparisons. Specifically, 863 DELs were identified in 'DTH' vs 'DHS' (365 upregulated and 498 downregulated); 2,486 DELs in 'HBAL' vs 'DHS' (628 upregulated and 1,858 downregulated); and 1,330 DELs in 'DTH' vs 'HBAL' (759 upregulated and 571 downregulated) (Fig. 6c).

Prediction of *cis*-regulatory targets of DELs indicated that 4,953 DELs may regulate 3,250 genes. Functional enrichment analysis showed that *cis* target genes of DELs in 'DTH' vs 'DHS' and 'DTH' vs 'HBAL' were enriched in the 'Metabolic pathways' and 'Biosynthesis of secondary metabolites' pathways. Notably, *cis* targets in the 'HBAL' vs 'DHS' were additionally enriched in the 'Flavonoid biosynthesis' pathway (Supplementary Figs S11−S13). Construction of DEL-flavonoid biosynthesis gene regulatory networks further identified 178 DELs as potential cis-regulators of 41 flavonoid biosynthesis genes, including the putative regulation of *GbCHS* by *LncNAT1* (Fig. 6d).

### LncNAT1 negatively regulates plant development and flavonoid biosynthesis by repressing *GbCHS*

**Figure 7:**
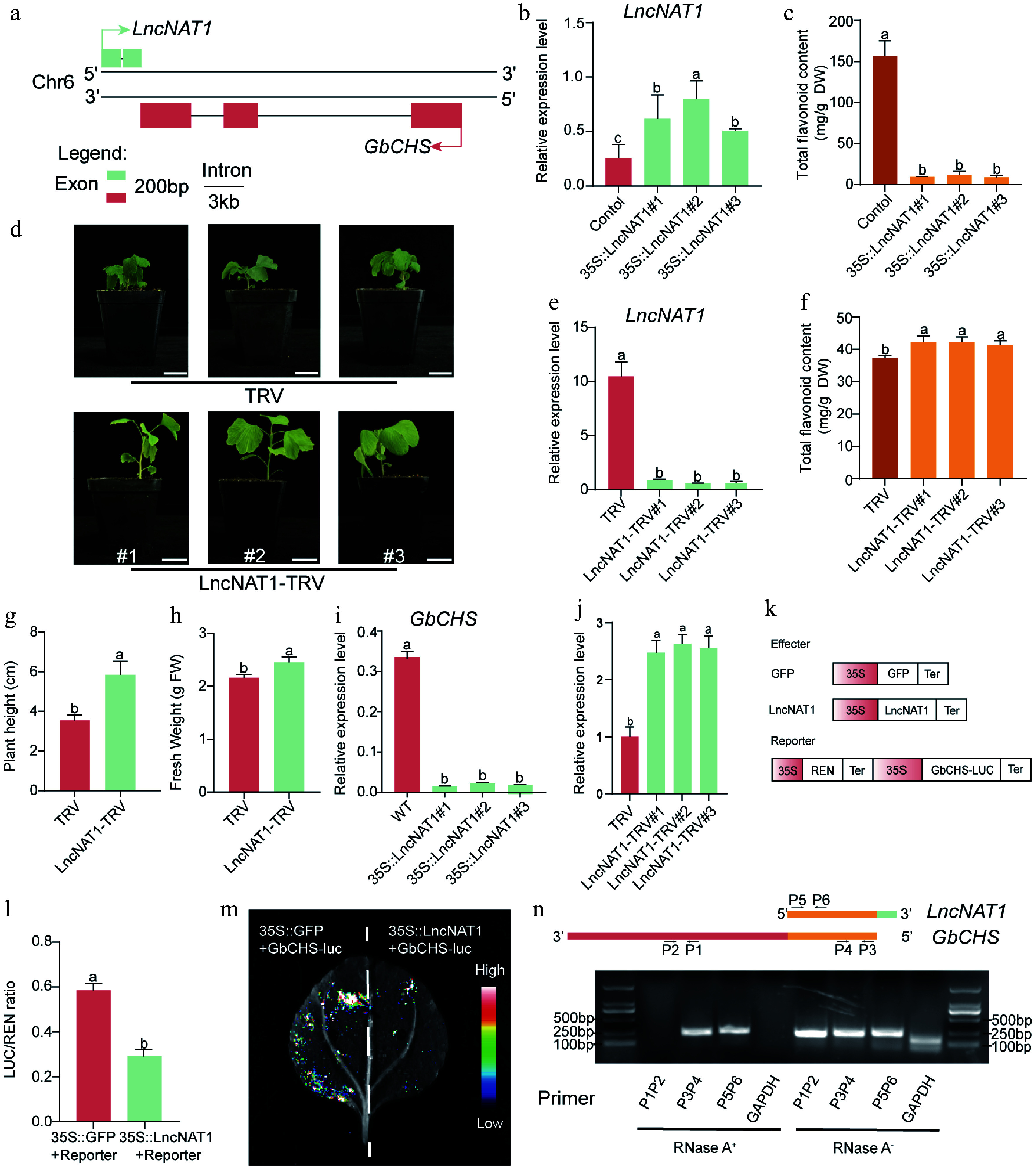
*LncNAT1* negatively regulates flavonoid biosynthesis and plant development by inhibiting *GbCHS.* (a) Genomic organization showing the antisense relationship between *LncNAT1* and *GbCHS*. (b) *LncNAT1* transcript levels, and (c) total flavonoid content in *LncNAT1*-overexpressing *G. biloba* calli. (d) Phenotypes of *LncNAT1*-silenced *G. bilob*a plants. Scale bars = 3 cm. (e) *LncNAT1* expression levels (f) and total flavonoid accumulation in *LncNAT1*-silenced *G. bilob*a plants. (g), (h) Comparison of plant height and fresh weight between *LncNAT1*-silenced plants and TRV control. *GbCHS* transcript levels in (i) *LncNAT1*-overexpressing calli and (j) *LncNAT1*-silenced plants. (k) Schematic of DLR vector. (l) DLR assay showing repression of *GbCHS* activity by *LncNAT1*. (m) Luciferase imaging assay verifies the regulatory relationship between *LncNAT1* and *GbCHS*. (n) RPA analysis of *LncNAT1*-*GbCHS* binding interaction. Different lowercase letters indicate significant differences (*p < 0.05*). Data represent mean ± SD of three biological replicates (*n* = 3).

Genomic locus analysis revealed that *LncNAT1* is transcribed antisense to *GbCHS* (*Gb_19002*), with partial sequence complementarity between the two transcripts (Fig. 7a, Supplementary Fig. S14). Sequence alignment further showed that *LncNAT1* shares only limited and fragmented similarity with other members of the *CHS* gene family (Supplementary Fig. S15). In contrast to *GbCHS* (*Gb_19002*), *LncNAT1* displayed its highest expression in the low-flavonoid cultivar 'DHS' and the lowest expression in the high-flavonoid cultivar 'HBAL' (Supplementary Fig. S16). To investigate the biological function of *LncNAT1*, 35S::LncNAT1 was overexpressed in *G. biloba* calli, and qRT-PCR confirmed strong transgene expression (Fig. 7b) accompanied by a dramatic reduction (92%−94%) in total flavonoid content relative to the empty vector control (Fig. 7c). Conversely, VIGS-mediated silencing of *LncNAT1* significantly decreased its transcript abundance and resulted in an 11%-14% increase in total flavonoids compared with the TRV control (Fig. 7d−f). In addition to its metabolic effects, *LncNAT1* silencing markedly promoted vegetative growth, with plant height and fresh weight increasing by 65% and 14%, respectively (Fig. 7g, h).

This study further examined whether *LncNAT1* regulates flavonoid biosynthesis by modulating *GbCHS* expression. It is revealed that overexpression of *LncNAT1* significantly suppressed *GbCHS* transcript levels, whereas *GbCHS* expression was markedly enhanced upon *LncNAT1* silencing (Fig. 7i, j). Furthermore, DLR assays demonstrated that co-expression of *LncNAT1* significantly reduced the LUC/REN ratio of *GbCHS* (Fig. 7k, l). These results demonstrate the inhibitory effect of *LncNAT1* on *GbCHS* at both the transcriptional and translational levels.

Given that *LncNAT1* is a type of lncNAT, it was tested to determine if it functions by duplex formation with *GbCHS* through RPA. RPA demonstrated that complementary regions of *LncNAT1* and *GbCHS* (P3/P4, P5/P6) remained resistant to RNase A digestion, whereas non-complementary regions (P1/P2) and the *GAPDH* control were almost completely degraded (Fig. 7m). These results indicate that *LncNAT1* represses *GbCHS* expression by forming a stable *LncNAT1*-*GbCHS* RNA duplex. Finally, expression analysis of all other *GbCHS* family members in the LncNAT1-silenced plant revealed that only *GbCHS* (*Gb_19002*) was significantly upregulated, while other paralogs remained unchanged (Supplementary Fig. S17). This specificity demonstrates that *LncNAT1* selectively targets *GbCHS* (*Gb_19002*) rather than broadly regulating the *CHS* gene family.

## Discussion

### Screening of *G. biloba* cultivars with high flavonoid content

Flavonoids constitute a major class of polyphenolic secondary metabolites that are widely distributed in plants^[10]^. *G. biloba* is recognized as an important medicinal species owing to its abundant flavonoid constituents^[28]^, which have been clinically validated for the treatment of cardiovascular and coronary heart diseases as well as neurodegenerative disorders^[48]^. To date, approximately 110 flavonoid compounds have been identified in *G. biloba* leaves^[49]^. More recent investigations have further revealed novel flavonoids such as biginkgosides A, B, C, and D^[50]^. Despite this chemical richness, the long juvenile period of *G. biloba* trees severely constrains conventional breeding programs aimed at improving flavonoid yield, resulting in a limited availability of elite, high-quality cultivars in the market. This study revealed a 3.45-fold variation in total flavonoid content among 26 *G. biloba* cultivars. 'HBAL' and 'DTH' accumulated high levels of eight major flavonoid subclasses, including biflavonoids and flavonols, whereas 'DHS' was characterized by elevated anthocyanin accumulation. These contrasting metabolic profiles suggest a competitive allocation of metabolic flux between the flavonoid and anthocyanin branches of the phenylpropanoid pathway, a phenomenon previously reported in other plant species where shared chalcone intermediates are differentially channeled toward distinct downstream products^[50]^. Together, these findings provide a scientific basis for the targeted selection of specialized *G. biloba* cultivars, such as high-flavonoid leaf-use types and high-anthocyanin ornamental types.

### *GbCHS* functions in flavonoid biosynthesis and plant development

*CHS* is the first committed and rate-limiting enzyme in the flavonoid biosynthetic pathway and is highly conserved across angiosperms^[51,52]^. For example, silencing *CiCHS* in citrus or knocking out *MdCHS* in apple leads to a marked reduction in flavonoid content, while in *A. thaliana*, *CHS* protein abundance is dynamically regulated through the ubiquitin-proteasome system, thereby modulating anthocyanin accumulation^[53−55]^. The result establishes *GbCHS* as a critical structural gene in the flavonoid biosynthetic pathway of *G. biloba*. The recombinant enzyme catalyzes the formation of naringenin chalcone from p-coumaroyl-CoA and malonyl-CoA *in vitro*, confirming its catalytic competence. *In vivo*, overexpression of *GbCHS* in *G. biloba* calli increased total flavonoid content by more than 49%, whereas VIGS-based silencing resulted in a reduction exceeding 35%. Beyond its metabolic function, accumulating evidence indicates that *CHS* exerts broad regulatory effects on plant growth and development. In petunia, maize, and tomato, *CHS* mutations or silencing disrupt flavonol accumulation in pollen, leading to defective pollen tube growth, male sterility, or abnormal fruit development^[56−58]^. More recently, silencing *CHS* in apple was shown to cause not only a near-complete loss of flavonoids but also pronounced vegetative defects, including shortened internodes, reduced leaf size, and diminished growth rates, which were associated with altered auxin transport dynamics^[54]^. This study extends these findings to *G. biloba*. Silencing of *GbCHS* resulted in reduced plant height, leaf number, and biomass, whereas heterologous overexpression of *GbCHS* in *A. thaliana* significantly enhanced primary root length, lateral root number, rosette leaf number, and biomass. These phenotypes are consistent with previous observations in *A. thaliana* flavonol-deficient synthesis mutants, where impaired flavonol biosynthesis disrupts root elongation, regulates inflorescence branching, and alters stomatal aperture^[59]^. Conversely, the enhanced growth traits observed in *GbCHS*-overexpressing *A. thaliana* plants further support the notion that flavonoids act as key modulators of developmental processes. Collectively, these results demonstrate that *GbCHS* functions not only as a central enzymatic hub in flavonoid biosynthesis but also as an important metabolic regulator coordinating flavonoid-dependent control of plant growth and development in *G. biloba* and beyond.

### Regulatory mechanism of the *LncNAT1*-*GbCHS* module: functional conservation and specificity of plant lncNATs

Natural antisense lncNATs represent an important class of regulatory RNAs in plants and typically exert their functions through sequence complementarity with their sense transcripts, thereby modulating gene expression at transcriptional and/or translational levels^[60,61]^. Several well-characterized lncNATs exemplify the mechanistic diversity of this regulatory mode. In petunia, the lncNAT *SHO* promotes the formation of double-stranded RNA and siRNA formation, leading to RNA interference-mediated degradation of the cytokinin biosynthesis gene *SHO*^[62]^. In rice, *cis*-*NATPHO1;2* associates with polysomal to enhance the translation efficiency of *PHO1;2*^[63]^. Meanwhile, in *A. thaliana*, the lncNAT *SVALKA* interferes with RNA polymerase II elongation, thereby repressing transcription of the cold-responsive gene *CBF1* through transcriptional collision^[64,65]^. Collectively, these studies highlight that plant lncNATs employ diverse regulatory strategies, including RNA interference, translational control, transcriptional interference, and chromatin-associated mechanisms.

The *LncNAT1*-*GbCHS* module identified in this study represents a distinct example of an antisense lncNAT directly targeting a structural gene. *LncNAT1* forms an RNA duplex with *GbCHS*, thereby repressing its expression and modulating both flavonoid biosynthesis and plant growth. Unlike most previously characterized plant lncNATs, which primarily regulate transcription factors or hormone-related genes, *LncNAT1* directly targets a core enzymatic gene in the flavonoid pathway, underscoring the specificity and precision of metabolic regulation in *G. biloba*. Furthermore, the RNA duplex-mediated mechanism *LncNAT1* differs fundamentally from that of rice *TL*, which regulates *OsMYB60* through histone modifications^[66]^, and *A. thaliana* *asHSFB2a*, which represses transcription factor expression^[67]^. Together, these differences highlight the species- and target-dependent functional diversity of plant lncNATs. This variety suggests that lncNAT-mediated regulatory networks are substantially more complex than currently appreciated, warranting systematic investigation across diverse species and metabolic contexts.

### Study limitations and future perspectives

Although this study clarifies the function and regulatory mechanism of the *LncNAT-GbCHS* module, several limitations remain. First, functional validation was primarily achieved through transient overexpression in *G. biloba* calli, VIGS-based gene silencing, and heterologous expression in *A. thaliana*, due to the lack of a stable genetic transformation system for *G. biloba*. Second, although the results demonstrate that *GbCHS* profoundly influences plant growth and development, the downstream molecular mechanisms, particularly the interplay between flavonoid accumulation and hormone signaling pathways such as auxin, need to be fully understood. Future research should therefore focus on two major directions. On the one hand, the establishment of a stable and efficient genetic transformation system for *G. biloba* will be essential for definitive functional validation of the *LncNAT1*-*GbCHS* module. On the other hand, integrative approaches combining transcriptomics, hormone profiling, and protein-protein or protein-metabolite interaction analyses will also play a critical role.

## Conclusions

**Figure 8:**
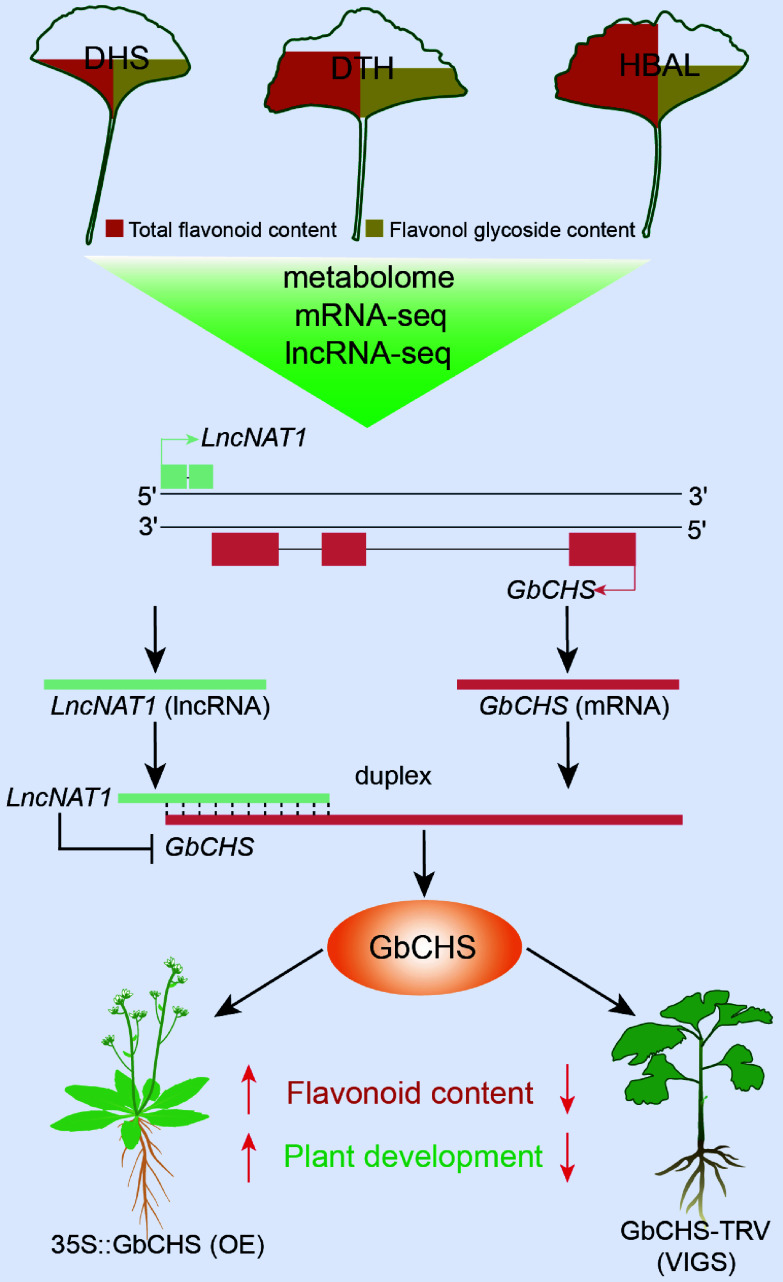
Working model of the *LncNAT1*-*GbCHS* regulatory module in flavonoid biosynthesis and plant development. Integrated analysis of metabolomic, RNA-seq, and lncRNA-seq datasets identified a DEL, *LncNAT1*, along with its potential target gene, *GbCHS*. Functional validation using dual-luciferase assays, RPA, and genetic transformation experiments demonstrated that *LncNAT1* directly binds to the *GbCHS* transcript to form an RNA duplex, thereby repressing *GbCHS* expression. This inhibition thereby negatively regulates flavonoid biosynthesis and plant growth and development in *G. biloba*.

This study reveals substantial natural variation in flavonoid accumulation among *G. biloba* cultivars, with 'HBAL' exhibiting the highest total flavonoid content. Integrated metabolomic and transcriptomic analyses identified 976 metabolites and 3,090 DEGs. Among them, *GbCHS* (*Gb_19002*) genes show a strong positive correlation with flavonoid accumulation. Functional assays confirmed that GbCHS catalyzes naringenin chalcone synthesis, and its overexpression significantly enhanced flavonoid accumulation in *G. biloba* calli and *A. thaliana*, whereas VIGS-mediated silencing caused a marked reduction. Moreover, overexpressing *GbCHS* positively regulates plant growth and development. Additionally, a potential *LncNAT-GbCHS* regulatory module was identified. *LncNAT1*, an lncNAT, negatively regulates flavonoid biosynthesis and plant development by directly inhibiting *GbCHS* through RNA duplex formation (Fig. 8). Collectively, these results uncover a novel lncNAT-mediated regulatory mechanism controlling flavonoid metabolism and growth in *G. biloba* plants and offer promising genetic targets for the coordinated improvement of flavonoid yield and growth traits in *G. biloba* breeding programs.

## SUPPLEMENTARY DATA

Supplementary data to this article can be found online.

## Data Availability

The ssRNA-seq data are available at the NCBI SRA database under accession number PRJNA1133634.
