# Supplementary material for: Unraveling the multifaceted roles of the LncNAT1-GbCHS module in Ginkgo biloba for flavonoid biosynthesis and plant development
Source: For Res (Fayettev). 2026 Mar 25;6:e006. doi: 10.48130/forres-0026-0006 (PMC13187911; doi:10.48130/forres-0026-0006)
Supplement: Supplementary file 1 — Supplementary data to this article can be found online. [file forres-0026-0006-S1.zip › 10.48130_forres-0026-0006-Suppl-FigureS1.pdf]

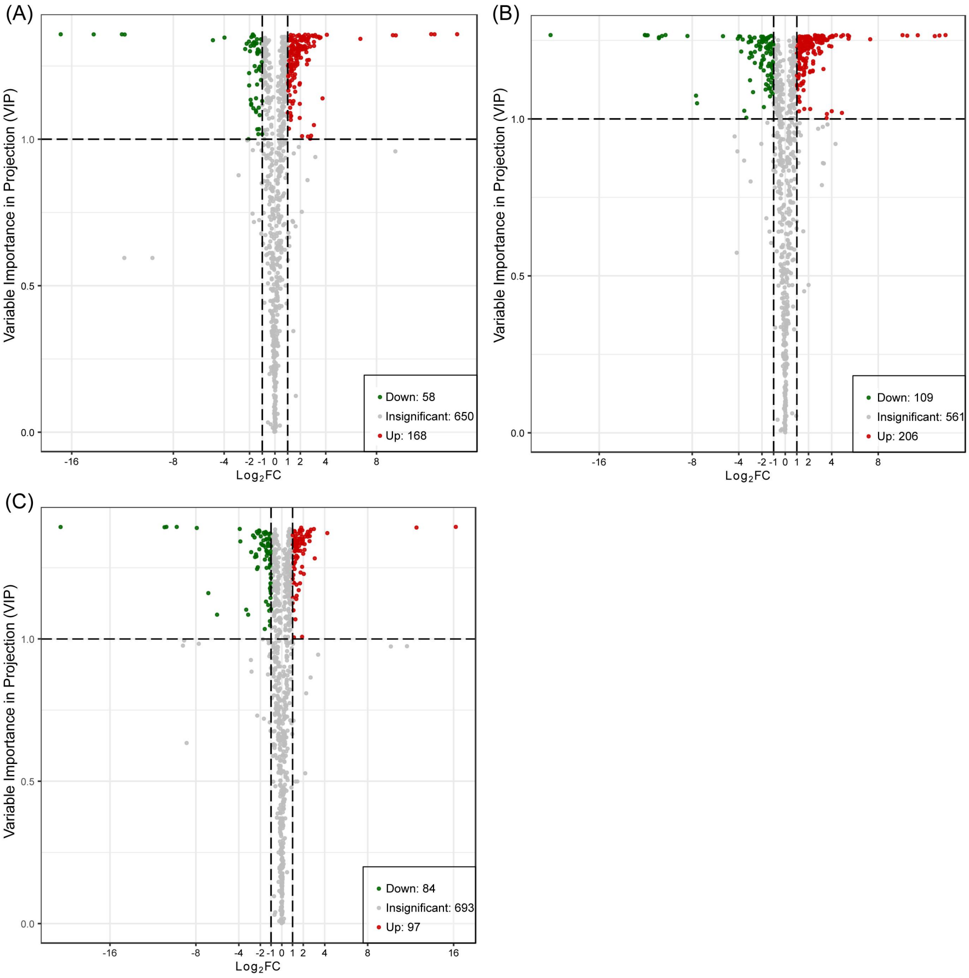

Fig. S1 Volcano plot of differentially accumulated metabolites (DAMs) in *G. biloba* leaves among different cultivar comparisons: (A)'DTH' vs. 'DHS,' (B) 'HBAL' vs. 'DHS,' and (C) 'DTH' vs. 'HBAL.'
