# Supplementary material for: Unraveling the multifaceted roles of the LncNAT1-GbCHS module in Ginkgo biloba for flavonoid biosynthesis and plant development
Source: For Res (Fayettev). 2026 Mar 25;6:e006. doi: 10.48130/forres-0026-0006 (PMC13187911; doi:10.48130/forres-0026-0006)
Supplement: Supplementary file 1 — Supplementary data to this article can be found online. [file forres-0026-0006-S1.zip › 10.48130_forres-0026-0006-Suppl-TableS1.pdf]

Supplemental Table. S1 Names and abbreviations of the *Ginkgo biloba* cultivars used in this study

Cultivars name	Cultivars name abbreviation
Dahuasui-1	DHS
Tiefu-3	TF
Taixing-4	TX
Dongtinghuang	DTH
Dongtingfoshou-3	DTFS
Jiangxi-2	JX
Dalongyan	DLY
Tancheng-262	TC
Guizhouzhengan-1	GZZA-1
Guizhouzhengan-2	GZZA-2
Guizhouzhengan-3	GZZA-3
Guizhouzhengan-4	GZZA-4
Daozhen-7	DZ
Zhejiangchangxing-1	ZJCX
Yuanling-13	YL
Jinzhui-5	JZ
Panxianchangbaiguo	PXCBG
Japantenjiulang	RBTJL
Hubeianlu1-4	HBAL
Hubei (Pengrisan)1-6	HBPRS
Hubeianlu-1-5	HBAL-2
Hubeianlu-1-6	HBAL-3
Dongbeidagushanshang	DBDGSS
Rugao (Longyan)	RGLY
Pixianmeihe	PXMH
Taianxiajin	TAXJ
