# Supplementary material for: Unraveling the multifaceted roles of the LncNAT1-GbCHS module in Ginkgo biloba for flavonoid biosynthesis and plant development
Source: For Res (Fayettev). 2026 Mar 25;6:e006. doi: 10.48130/forres-0026-0006 (PMC13187911; doi:10.48130/forres-0026-0006)
Supplement: Supplementary file 1 — Supplementary data to this article can be found online. [file forres-0026-0006-S1.zip › 10.48130_forres-0026-0006-Suppl-TableS2.pdf]

Supplemental Table. S2 Primer sequences used in the experiment.

Gene	Primer sequences
LncNAT1	F:TCGTCAGCTGGTAGTCGG
(TCONS_00200861)	R:AGCAGATTGTAGTCCAGAATGCC
GbCHS (Gb_19002)	F:ATGCCTGCAGGAGCGATGAA
	R:ATTGTTGCAGGGAACGCTTCTT
35S::LncNAT1	F:gaccccgggggtaccggatccTCGTCAGCTGGTAGTCGGCC
	R:tttacccatgaattcggatccAGCAGATTGTAGTCCAGAATGCC
35S::GbCHS	F:gaccccgggggtaccggatccATGCCTGCAGGAGCGATGA
	R:tttacccatgaattcggatccATTGTTGCAGGGAACGCTTC
pACT2-GbCHS-LUC	F:aaactgcagcccgggggatccATGCCTGCAGGAGCGATG
	R:ctctagaactagtgtggatccATTGTTGCAGGGAACGCTTC
pET-32a-GbCHS	F:gccatggctgatatcggatccATGCCTGCAGGAGCGATG
	R:tgcggccgcaagcttgtcgacATTGTTGCAGGGAACGCTTC
GAPDH	F:CTGCCAAGGCTGTAGGTAAGG
	R:TCAGATTCCTCCTTGATGGCG
LncNAT1-qRT-PCR	F:CCTCCACCACCACCATGTCCTG
	R:GCATGTGTGACAAGTCAGCGATAAAG
GbCHS-qRT-PCR	F: ATGCCTGCAGGAGCGATGAA
	R: AAGTAGTAATCGGGATAATTG
LncNAT1-VIGS	F:gtgagtaaggttaccgaattcTCGTCAGCTGGTAGTCGGCC
	R:gagacgcgtgagctcggtaccTCAAGCGCATGTGCGACA
GbCHS-VIGS	F:gtgagtaaggttaccgaattcTGGTTTGCAGTGAAATAACGGC
	R:gagacgcgtgagctcggtaccGGACGTCCTTGAGGAGGTGG
P3	ATGCCTGCAGGAGCGATGG
P4	TATCTTTTCTTTATCGATGACTTGTCG
P1	TGGTTTGCAGTGAAATAACGGC
P2	GGACGTCCTTGAGGAGGTGG
P5	TCGTCAGCTGGTAGTCGGCC
P6	CATGTACTTGACGGAGGAGATACTG
