# Supplementary material for: Unraveling the multifaceted roles of the LncNAT1-GbCHS module in Ginkgo biloba for flavonoid biosynthesis and plant development
Source: For Res (Fayettev). 2026 Mar 25;6:e006. doi: 10.48130/forres-0026-0006 (PMC13187911; doi:10.48130/forres-0026-0006)
Supplement: Supplementary file 1 — Supplementary data to this article can be found online. [file forres-0026-0006-S1.zip › 10.48130_forres-0026-0006-Suppl-TableS3.pdf]

Supplemental Table. S3 Summary statistics of metabolite classes and numbers identified by widely targeted metabolomics.

Type	Number	Percentage
All	876	100%
Flavonoids	200	22.8%
Phenolic acids	159	18.2%
Lipids	150	17.1%
Amino acids and derivatives	81	9.2%
Others	74	8.4%
Organic acids	65	7.4%
Nucleotides and derivatives	46	5.3%
Alkaloids	45	5.1%
Lignans and Coumarins	23	2.6%
Tannins	21	2.4%
Terpenoids	12	1.4%
