# Supplementary figures and images for: Unraveling the multifaceted roles of the LncNAT1-GbCHS module in Ginkgo biloba for flavonoid biosynthesis and plant development
Source: For Res (Fayettev). 2026 Mar 25;6:e006. doi: 10.48130/forres-0026-0006 (PMC13187911; doi:10.48130/forres-0026-0006)

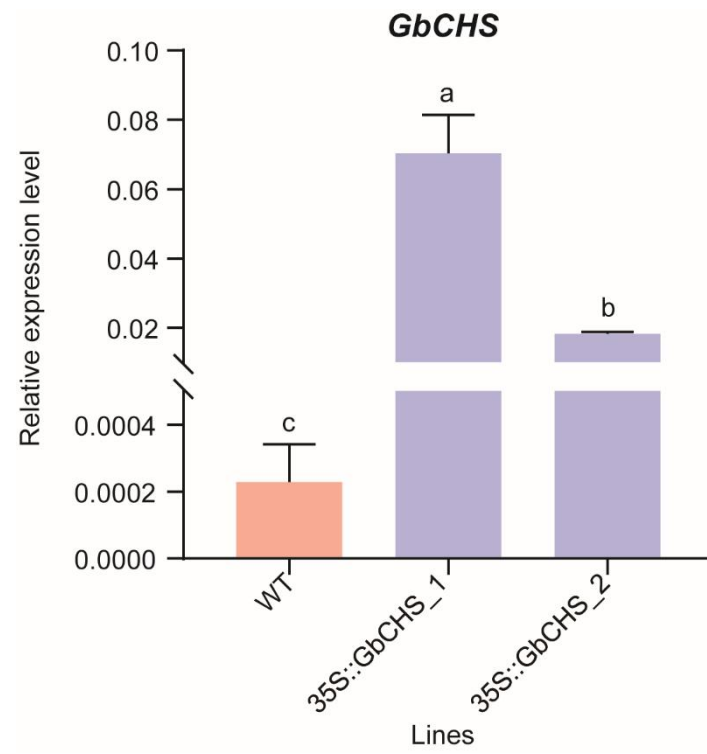

**Fig. S10** Expression levels of *GbCHS* in the *GbCHS*-overexpressing *A. thaliana* lines.

Supplement: Supplementary file 1 — Supplementary data to this article can be found online. [file forres-0026-0006-S1.zip › 10.48130_forres-0026-0006-Suppl-FigureS10.pdf]

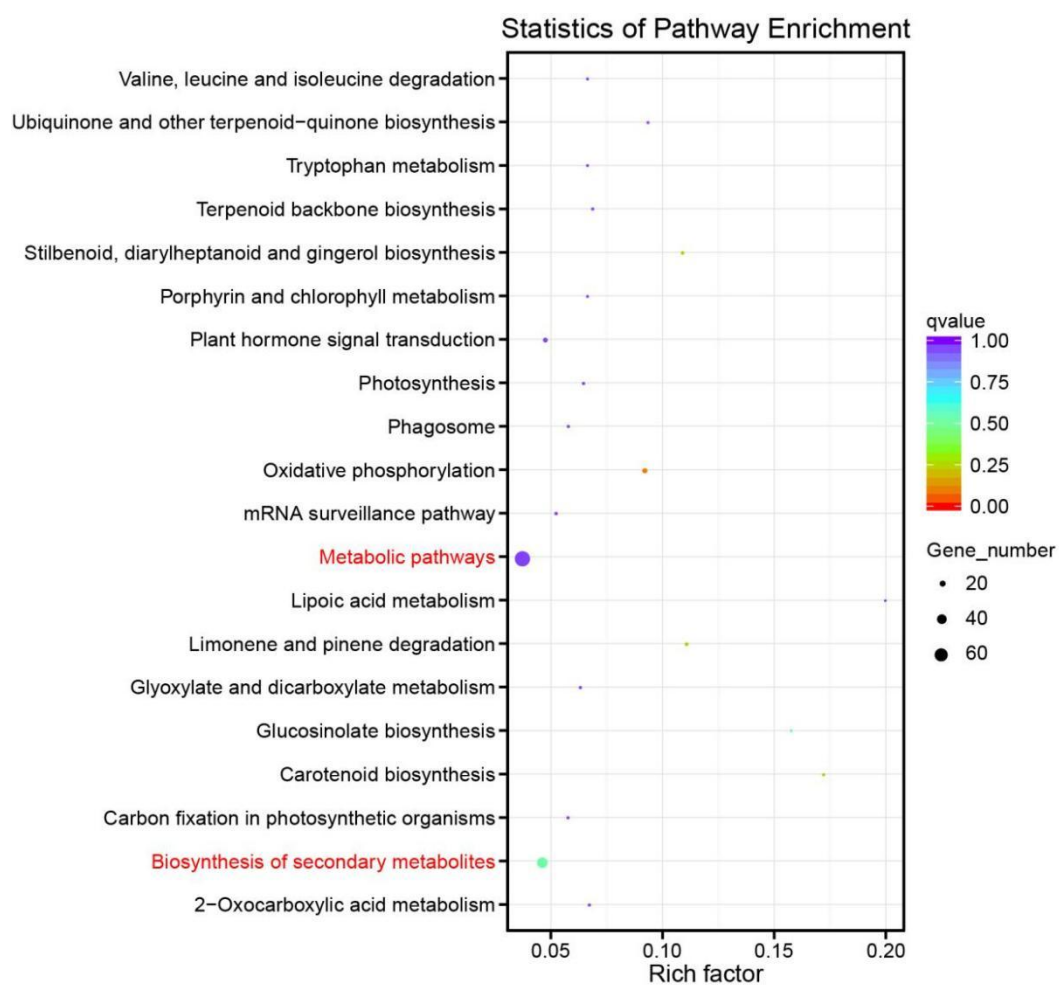

**Fig. S11** KEGG enrichment analysis of *cis*-regulated target genes of DELs in 'DTH' vs. 'DHS' comparison.

Supplement: Supplementary file 1 — Supplementary data to this article can be found online. [file forres-0026-0006-S1.zip › 10.48130_forres-0026-0006-Suppl-FigureS11.pdf]

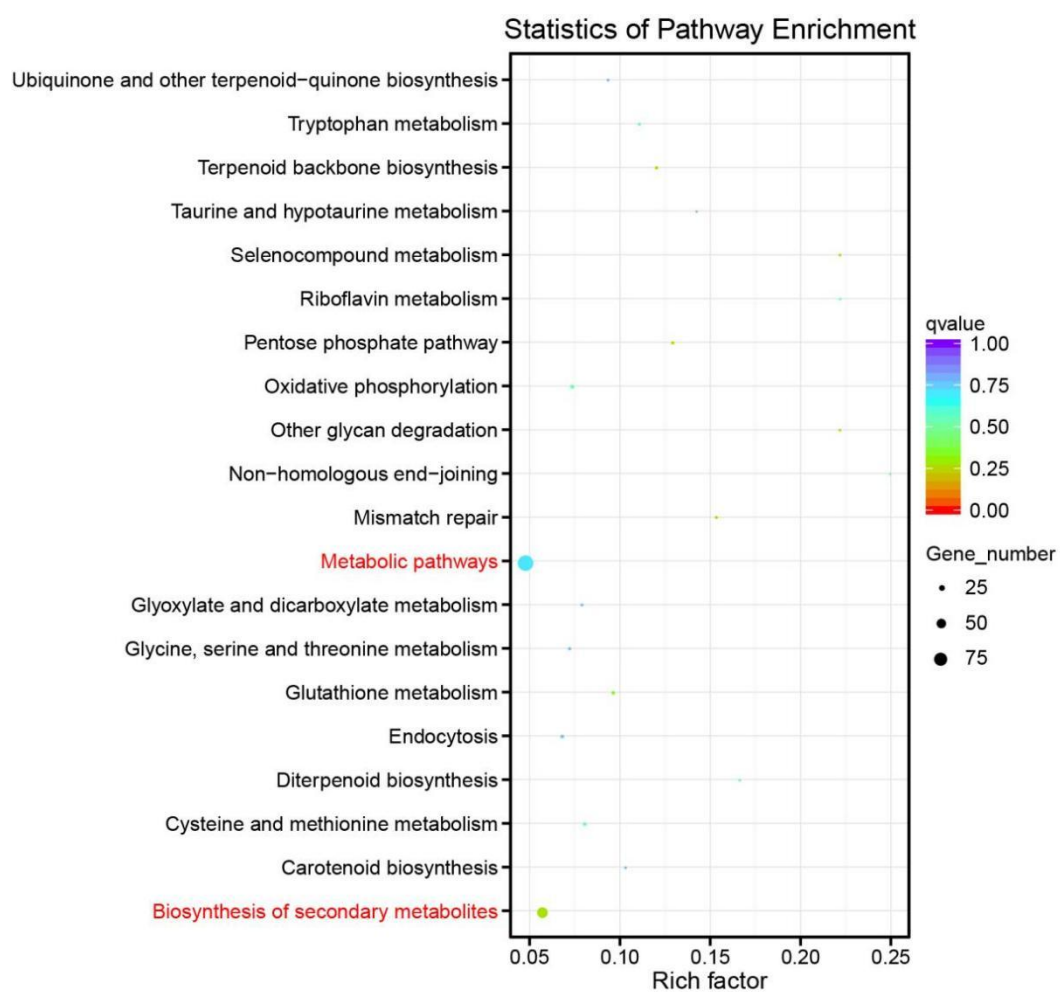

**Fig. S12** KEGG enrichment analysis of *cis*-regulated target genes of DELs in 'DTH' vs. 'HBAL.'

Supplement: Supplementary file 1 — Supplementary data to this article can be found online. [file forres-0026-0006-S1.zip › 10.48130_forres-0026-0006-Suppl-FigureS12.pdf]

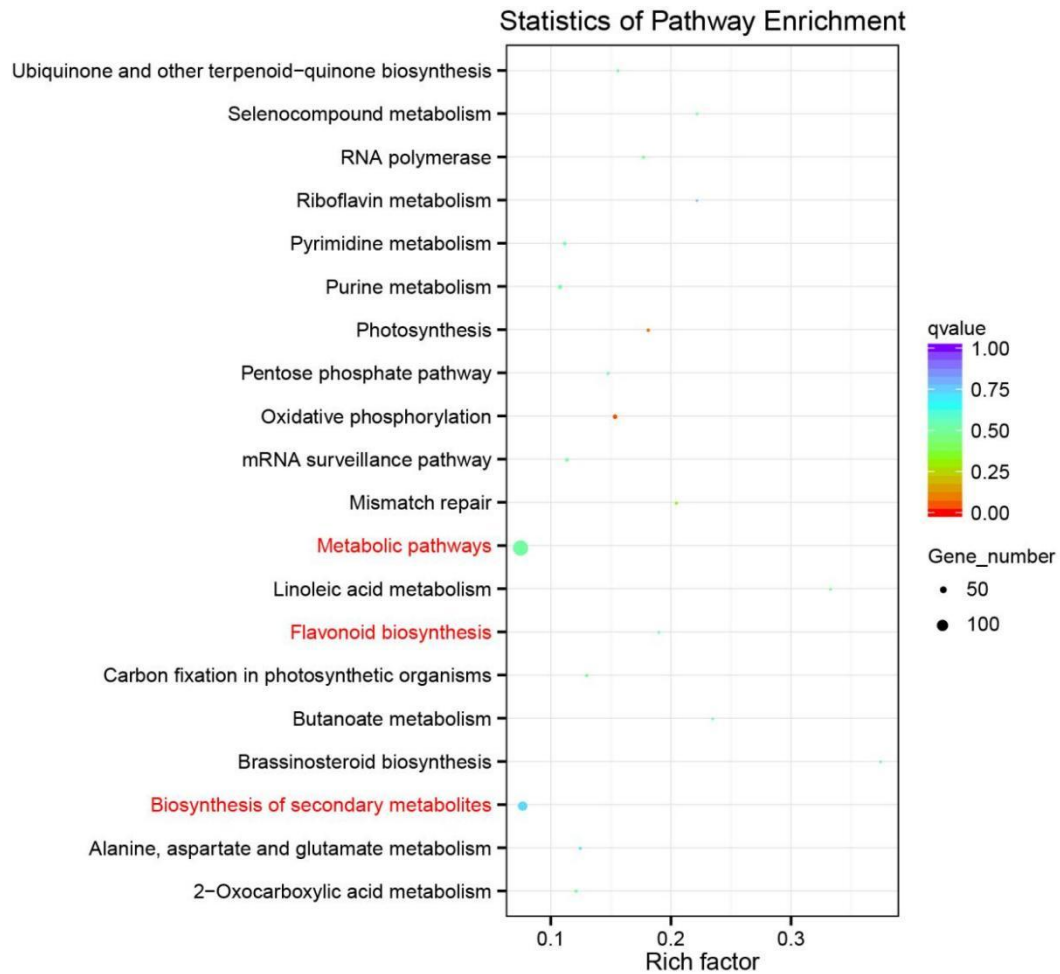

**Fig. S13** KEGG enrichment analysis of *cis*-regulated target genes of DELs in 'HBAL' vs. 'DHS' comparison.

Supplement: Supplementary file 1 — Supplementary data to this article can be found online. [file forres-0026-0006-S1.zip › 10.48130_forres-0026-0006-Suppl-FigureS13.pdf]

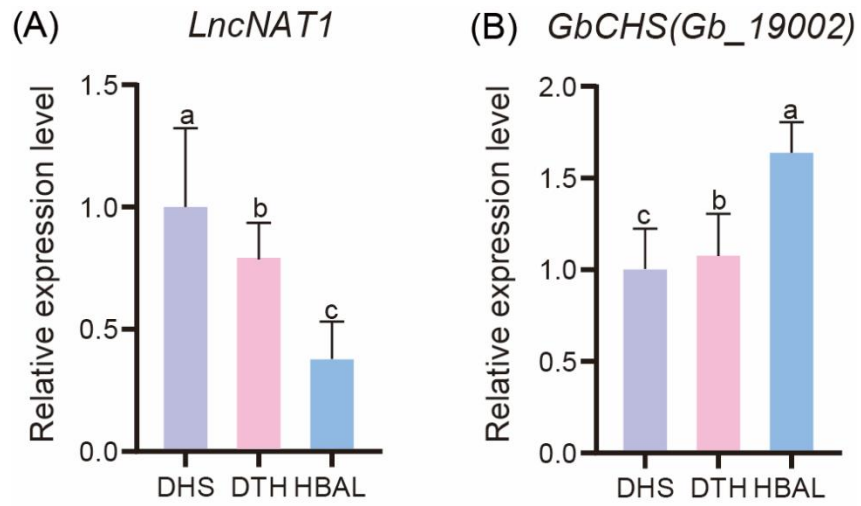

**Fig. S16** Expression patterns of *LncNAT1* and *GbCHS* across different cultivars.

Supplement: Supplementary file 1 — Supplementary data to this article can be found online. [file forres-0026-0006-S1.zip › 10.48130_forres-0026-0006-Suppl-FigureS16.pdf]

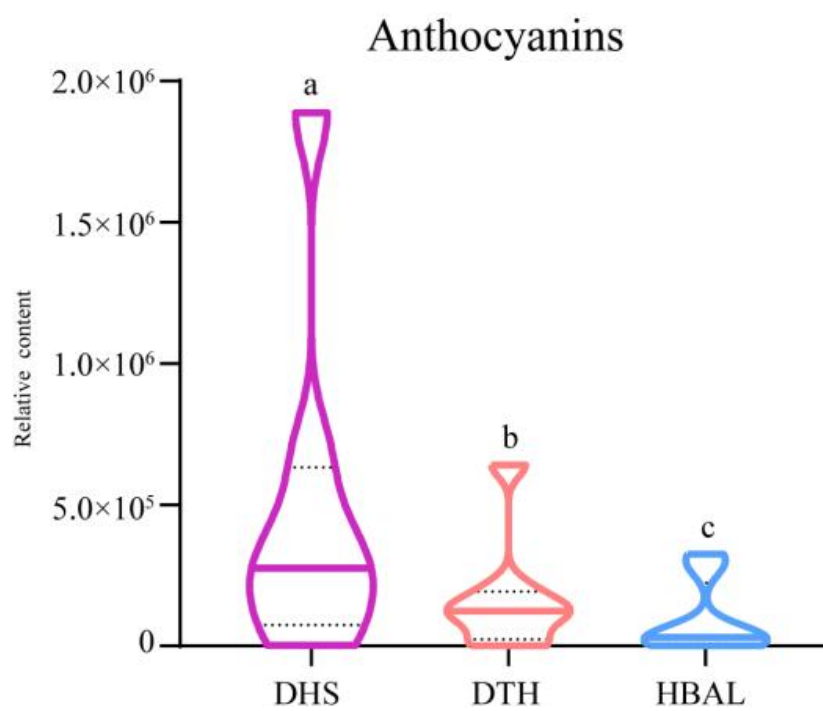

**Fig. S2** Relative content of anthocyanins in the leaves of the three selected cultivars.

Supplement: Supplementary file 1 — Supplementary data to this article can be found online. [file forres-0026-0006-S1.zip › 10.48130_forres-0026-0006-Suppl-FigureS2.pdf]

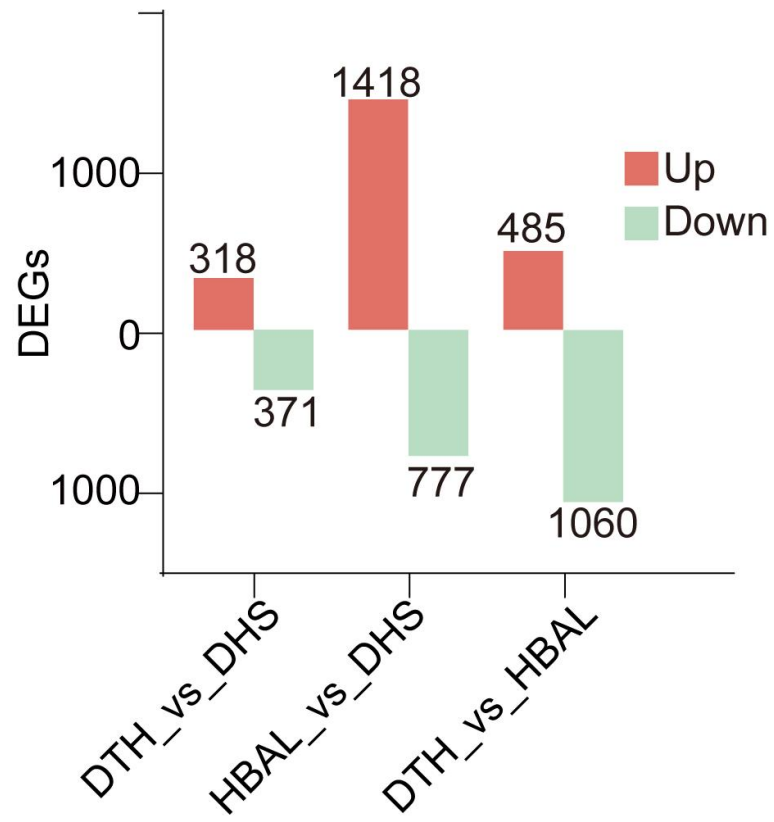

**Fig. S3** Numbers of differentially expressed genes (DEGs).

Supplement: Supplementary file 1 — Supplementary data to this article can be found online. [file forres-0026-0006-S1.zip › 10.48130_forres-0026-0006-Suppl-FigureS3.pdf]

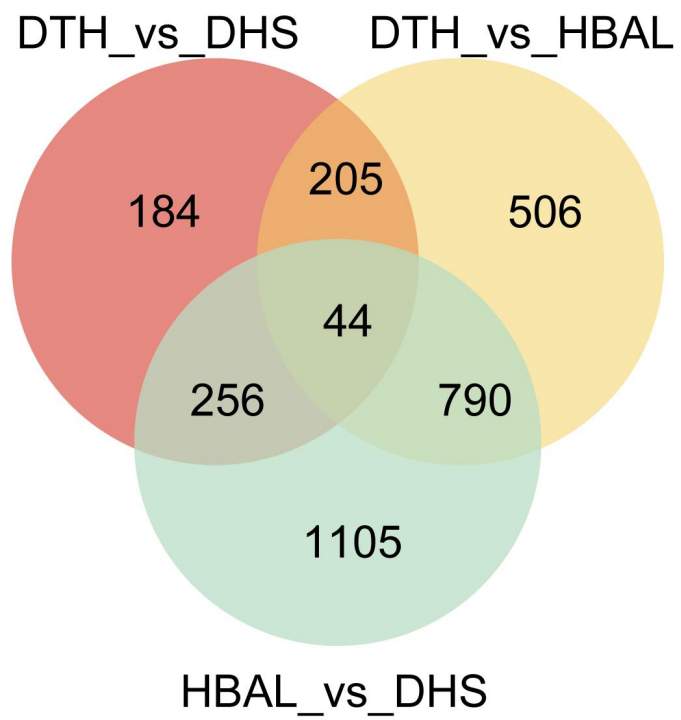

**Fig. S4** Venn diagram showing shared and unique DEGs across different comparison groups.

Supplement: Supplementary file 1 — Supplementary data to this article can be found online. [file forres-0026-0006-S1.zip › 10.48130_forres-0026-0006-Suppl-FigureS4.pdf]

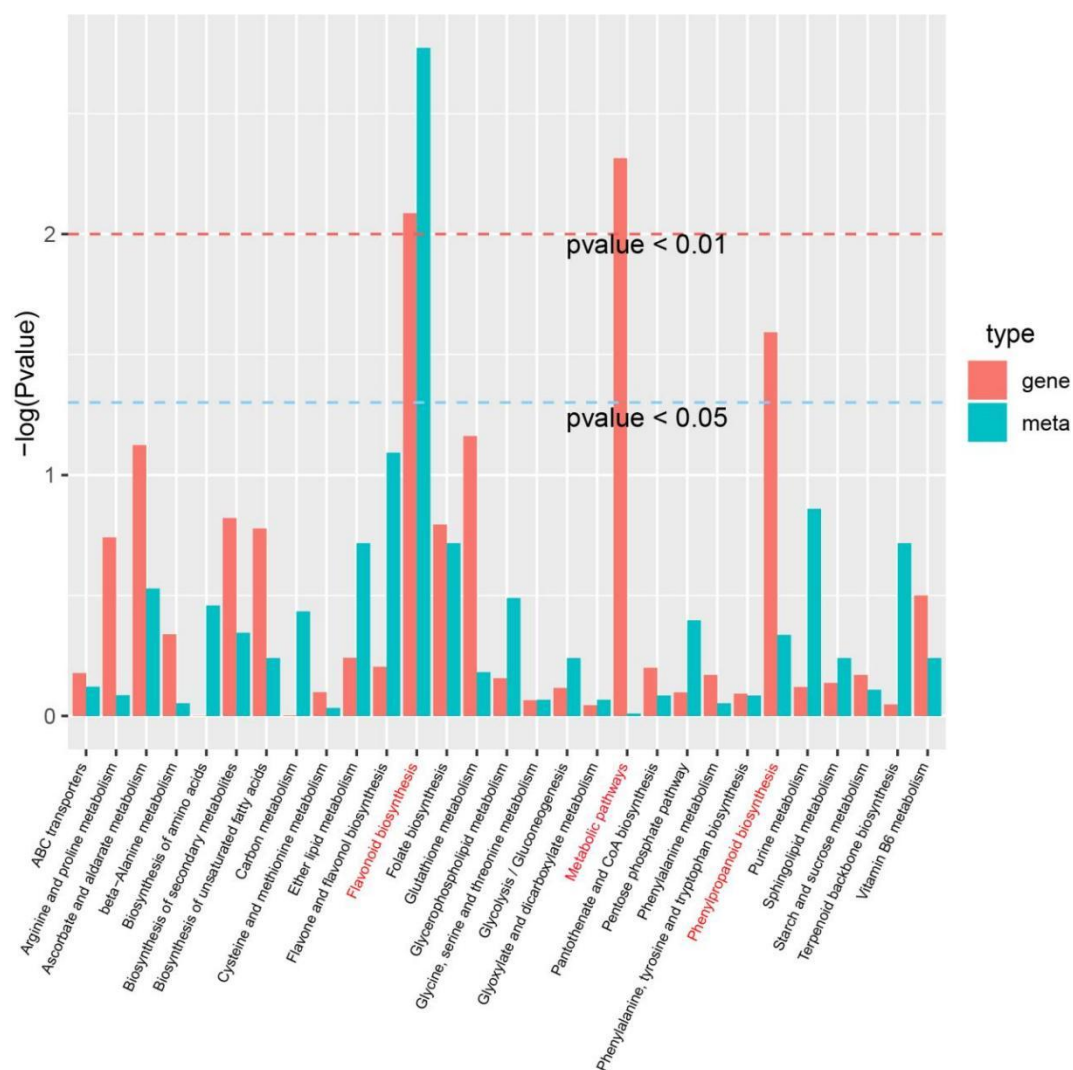

**Fig. S5** KEGG enrichment analysis of DAMs and DEGs in 'DTH' vs. 'DHS' comparison.

Supplement: Supplementary file 1 — Supplementary data to this article can be found online. [file forres-0026-0006-S1.zip › 10.48130_forres-0026-0006-Suppl-FigureS5.pdf]

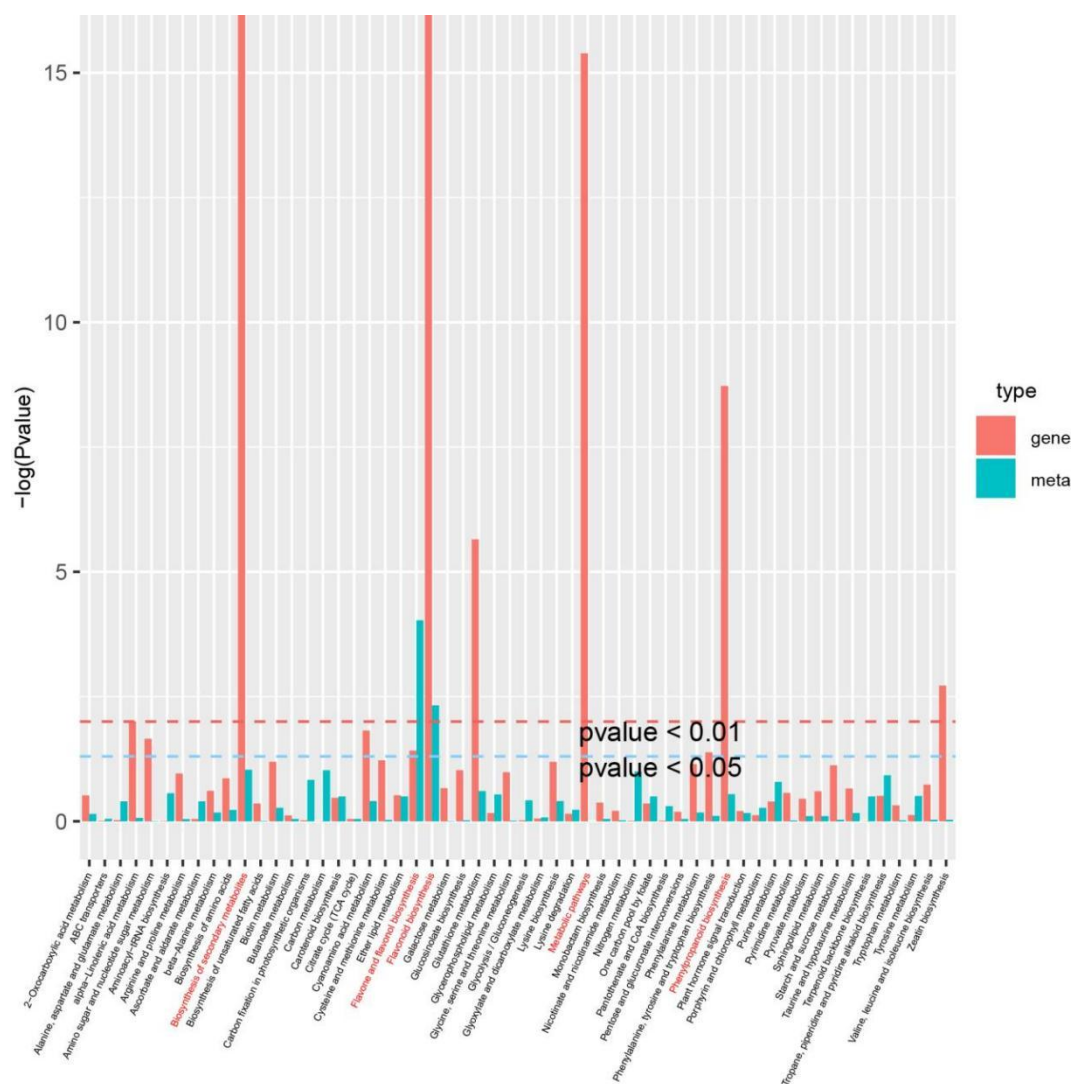

**Fig. S6** KEGG enrichment analysis of DAMs and DEGs in 'HBAL' vs. 'DHS' comparison.

Supplement: Supplementary file 1 — Supplementary data to this article can be found online. [file forres-0026-0006-S1.zip › 10.48130_forres-0026-0006-Suppl-FigureS6.pdf]

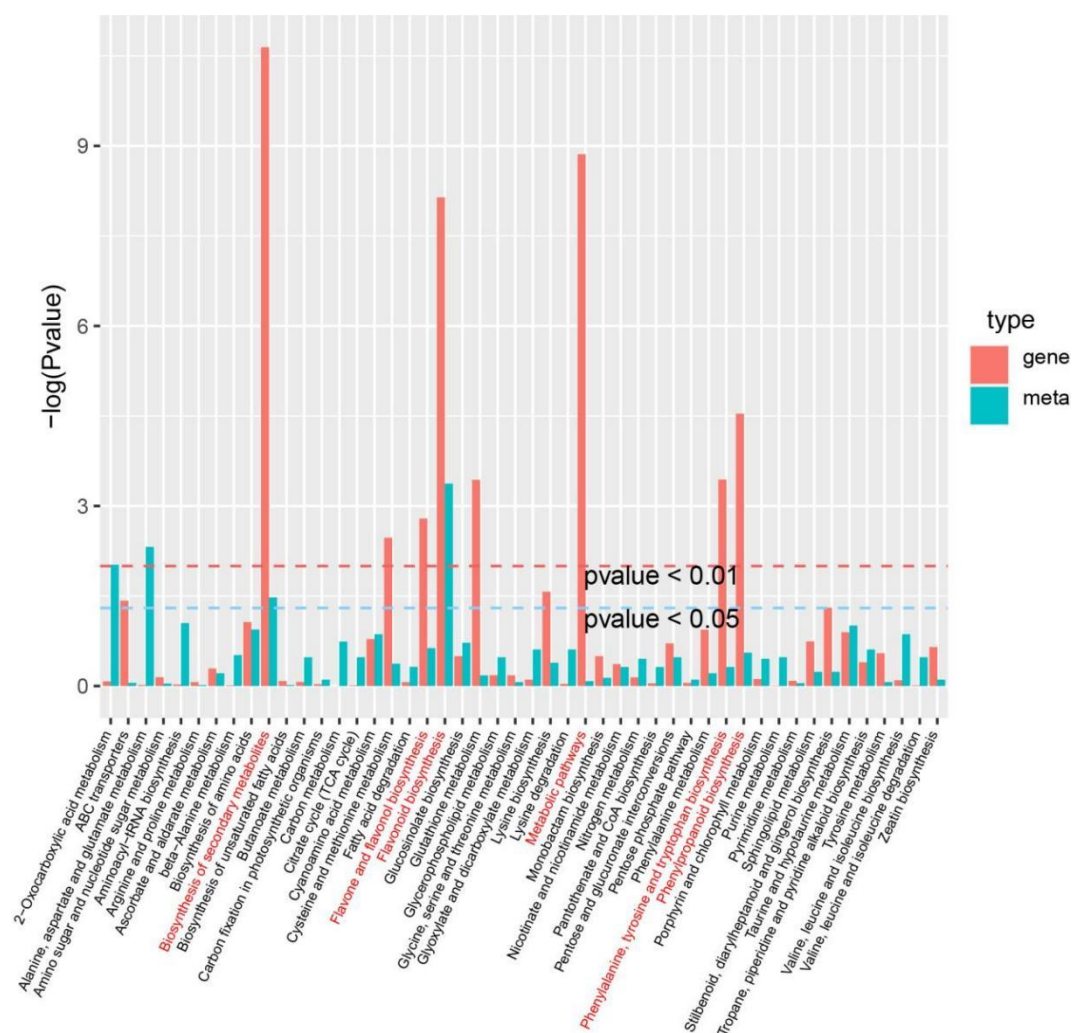

**Fig. S7** KEGG enrichment analysis of DAMs and DEGs in 'DTH' vs. 'HBAL'.

Supplement: Supplementary file 1 — Supplementary data to this article can be found online. [file forres-0026-0006-S1.zip › 10.48130_forres-0026-0006-Suppl-FigureS7.pdf]
